# Beyond survival to domination: *Brucella*’s multilayered strategies for evading host immune responses

**DOI:** 10.3389/fmicb.2025.1608617

**Published:** 2025-06-18

**Authors:** Zhimeng Wei, Shuai Zhang, Xingya Wang, Jie Bai, Hui Wang, Yuanchao Yang, Jingbo Zhai

**Affiliations:** ^1^School of Basic Medical Sciences, Inner Mongolia Minzu University, Tongliao, China; ^2^Department of Clinical Laboratory, Keerqin District First People’s Hospital, Tongliao, China; ^3^Department of Polyclinics, Tongliao City Center for Disease Control and Prevention, Tongliao, China; ^4^Brucellosis Prevention and Treatment Engineering Research Center of Inner Mongolia Autonomous Region, Tongliao, China; ^5^Key Laboratory of Zoonose Prevention and Control at Universities of Inner Mongolia Autonomous Region, Tongliao, China

**Keywords:** *Brucella*, virulence factors, immune escape, innate immunity, inflammasomes, ferroptosis

## Abstract

*Brucella* is an intracellular parasitic bacterium with a wide host range. It can infect terrestrial mammals, including domestic animals such as cattle and sheep, as well as wild animals like elk and bison. It also infects marine mammals, and amphibians. These diverse hosts form the basis for the classification of *Brucella* into different species. It can invade multiple cell types, including human cells such as monocytes/macrophages, dendritic cells (DCs), and trophoblasts; primary animal cells such as murine and bovine macrophages, and canine trophoblasts; and established cell lines such as HeLa and Vero cells. Among these, macrophages, DCs, and trophoblasts are the main target cells. *Brucella* employs a variety of strategies to evade host defenses: (1) obstruction of pattern recognition receptors; (2) formation of replicative *Brucella*-containing vacuoles following entry into host cells; (3) suppression of innate immunity through manipulation of autophagy, endoplasmic reticulum stress, inflammasomes, pyroptosis, apoptosis, ferroptosis, and pathways including cGAS-STING; and (4) inhibition of adaptive immunity through reduced antigen presentation. Compromised innate and adaptive immunity allows *Brucella* to replicate and survive within host cells, leading to chronic infections that are difficult to eradicate. Notably, *Brucella* suppresses host immunity by producing virulence factors that inhibit cytokine release and antigen presentation, and that interfere with critical signaling pathways such as programed cell death, ultimately downregulating both innate and adaptive immune responses. Collectively, these features have made the development of treatments and vaccines for brucellosis particularly challenging. While a better understanding of virulence factors is key to the effective prevention and control of brucellosis, many pathogenic mechanisms remain unclear. In this systematic review, we focus on the interactions between *Brucella* and the host immune system. Specifically, we examine the roles of the following factors in *Brucella* infection: lipopolysaccharides, flagella, the type IV secretion system (T4SS), effector proteins secreted by the T4SSs and non-T4SS, outer membrane proteins, phosphatidylcholine, mechanisms of intracellular survival, pathogen-associated molecular patterns, pattern recognition receptors, subversion of selective autophagy, endoplasmic reticulum stress pathways, inflammasomes, pyroptosis, apoptosis, ferroptosis, and the cGAS-STING pathway. We anticipate that this overview will offer new insights for research and development into drugs and vaccines for brucellosis.

## 1 Introduction

Brucellosis, caused by various *Brucella* species, is a global zoonotic disease affecting over 170 countries, with 1.6–2.1 million new human cases reported annually. In China, it is categorized as a class B infectious disease (Yang H. et al., 2020; Qureshi et al., 2023). *Brucella* species are host-specific, and include *B. abortus* in cattle, *B. melitensis* in goats and camels, *B. suis* in pigs, *B. ovis* in sheep, *B. abortus* in camels, elk, and bison, *B. canis* in dogs, *B. neotomae* and *B. microti* in rodents, *B. papionis* in monkeys, *B. pinnipedialis* and *B. ceti* in marine mammals (e.g., seals, dolphins, and whales), and *B. inopinata* in amphibians. *Brucella* can infect a variety of terrestrial and marine mammals, among which *B. melitensis* is the most pathogenic and invasive species for humans, followed by *B. abortus*, *B. suis*, and *B. canis* (Qin et al., 2008; Celli, 2019; Bialer et al., 2020; Gunes and Dogan, 2013; Waktole et al., 2022).

*Brucella* infects humans through several routes: (1) mucous membranes of the gastrointestinal tract, primarily following consumption of contaminated unpasteurized dairy or undercooked meats; (2) mucous membranes of the respiratory tract, primarily following inhalation of aerosolized bacteria; and (3) other mucous membranes and damaged skin, primarily following exposure to infected animals (Atluri et al., 2011; Yu et al., 2024). For humans, *Brucella* infection can be either asymptomatic or symptomatic, with an incubation period of 1–5 weeks, depending on the virulence of the pathogen. Based on the duration of symptoms, brucellosis is typically classified as acute (≤ 8 weeks), subacute (8–52 weeks), or chronic (> 1 year) (Deng Y. et al., 2019). Because of its intracellular parasitism and immune evasion features, *Brucella* infection can easily change from an acute condition to a chronic, refractory disease with multiple complications. These include the following: (1) skeletal infections that cause arthritis and joint pain, which can potentially lead to disability; (2) genitourinary system infections, leading to epididymis-orchitis in males and miscarriage in females; and (3) infections of other systems, with symptoms that include weakness, muscle pain, flu-like symptoms, undulant fever, endocarditis, hepatitis, meningitis, and neurological issues (Atluri et al., 2011; Yu et al., 2024; Głowacka et al., 2018; Khairullah et al., 2024). People of all ages and both sexes are at risk for brucellosis, with unreported symptomatic cases estimated to be 10 times higher than the reported cases (Khairullah et al., 2024). Despite its low case-fatality rate, brucellosis can significantly impact quality of life. In animal hosts, *Brucella* primarily invades reproductive systems (e.g., placenta, mammary glands, and epididymis), causing infertility, miscarriage, weight loss, and reduced milk production, seriously impacting animal husbandry (Bialer et al., 2020; Atluri et al., 2011).

Due to its multiple features, *Brucella* is considered as a biological weapon. As a Gram-negative facultative intracellular bacterium, it requires as few as 10–100 organisms to efficiently infect a host via aerosol transmission. It can also spread through various routes, including contact with infected animals and ingestion of contaminated food. *Brucella* is easy to cultivate, store, and transport from biological samples, and it remains stable in environments such as dairy products and soil. Infection can lead to chronic brucellosis, which is characterized by prolonged fever and organ damage. Although the direct mortality rate is low, the disease can place a heavy burden on healthcare systems and cause widespread public panic. As a zoonotic pathogen, it also poses a threat to livestock and may lead to agricultural security crises. In the past, *Brucella* was considered as a candidate for biological weapon development. Due to its high infectivity, ease of acquisition, and potential socio-economic impact, the U.S. Centers for Disease Control and Prevention (CDC) has classified it as a category B bioterrorism agent (Doganay and Doganay, 2013).

Currently, several live-attenuated vaccines have been approved for the prevention of brucellosis in animals, including *B. melitensis* Rev. 1, *B. abortus* RB51, and *B. abortus* S19. *B. melitensis* Rev. 1 and *B. abortus* S19 are both smooth live-attenuated vaccines and are considered the two traditional vaccines for the prevention and control of animal brucellosis. They also serve as important reference standards for evaluating novel vaccine candidates. *B. melitensis* Rev. 1 is currently the most effective vaccine for goats and sheep. *B. abortus* S19 is used to prevent *Brucella* infection in cattle. *B. abortus* RB51 is a genetically engineered, rough-type live-attenuated vaccine developed for the control of bovine brucellosis. It is considered a promising candidate for replacing the traditional S19 vaccine, particularly in situations where its lack of O antigen helps avoid serological interference (Heidary et al., 2022). Although these vaccines have shown some effectiveness in controlling the spread of brucellosis among animals, their efficacy varies depending on host species, vaccine dosage, and the route of administration (Yang et al., 2013; Wang and Wu, 2013). Meanwhile, they have clear limitations. In some cases, they may induce abortion in vaccinated animals, and due to their relatively high virulence, they are not suitable for human use (Perkins et al., 2010). To date, no brucellosis vaccine has been approved for use in humans (Wells et al., 2022). Vaccine development remains challenging, and most current strategies have achieved limited success. The pathogenesis of *Brucella*, including its mechanisms of immune evasion and persistent infection, is still not fully understood. Scare pathogen-associated molecular patterns (PAMPs) with strong immunogenicity in *Brucella* significantly reduces the availability of effective targets for vaccine development. Moreover, the high genetic variability and diversity of *Brucella* strains make it difficult for vaccines based on a single strain or antigen to provide broad and effective protection against multiple variants. In practical vaccine development, traditional live attenuated vaccines can induce long-lasting antibody responses and provide relatively strong protection. However, they also present notable challenges, including interference with serological diagnostics, potential induction of antibiotic resistance. Recently, various novel vaccine strategies have emerged. Subunit vaccines offer advantages such as non-infectivity and potential cross-protection, but they often suffer from weak antigenicity, poor stability, and a short half-life. DNA vaccines can elicit immune responses but generally fail to confer long-term protection. Vector-based vaccines, which introduce antigen-encoding genes into attenuated bacteria or viruses to enhance immunogenicity, may carry unforeseen risks due to differences from native proteins. Notably, live attenuated vaccines with virulence gene deletions have shown significantly improved safety profiles. These vaccines can promote T cell proliferation, pro-inflammatory cytokine expression, and antibody production, making them strong candidates for human use. However, concerns about possible reversion to virulence and the development of antibiotic resistance still need to be addressed (Yang et al., 2024). Currently, human brucellosis is treated with a combination of doxycycline and streptomycin (Zheng et al., 2024). However, the failure and recurrence rates of brucellosis treatment remain high. Therefore, gaining a deeper understanding of the pathogenesis and host immune responses to brucellosis is crucial.

## 2 Immune evasion by *Brucella*

*Brucella*, as a Gram-negative facultative intracellular bacterium, can cross mucosal barriers (e.g., in the gastrointestinal, respiratory, and genital tracts, and the conjunctiva), penetrate damaged skin, invade immune cells (e.g., macrophages and dendritic cells [DCs]), and disseminate systemically via the lymphatic and blood circulation. Subsequently, *Brucella* colonizes non-phagocytic and phagocytic cells in a variety of tissues and organs, enabling the bacteria to evade the immune system and replicate within cells. Ultimately, the pathogen exits these cells and establishes persistent infections in bodily tissues (de Figueiredo et al., 2015; Atluri et al., 2011; Khairullah et al., 2024; Myeni et al., 2013). Initially, most studies on *Brucella* were conducted using murine macrophage cell lines, bovine macrophages, human monocytes, and widely used non-phagocytic cell lines, such as HeLa and Vero cells. However, further *in vitro* research has shown that *Brucella* can infect and replicate in a variety of human cell types, including osteoblasts, synovial cells, trophoblasts, endothelial cells, lung epithelial cells, dendritic cells, and hepatocytes (Bialer et al., 2020). Additionally, *Brucella* can also infect and replicate in murine alveolar macrophages, canine trophoblasts and phagocytes, as well as in ovine testicular cells (Bialer et al., 2020). Notably, macrophages, DCs, and trophoblasts have been identified as the primary targets for *Brucella* infections (Jansen et al., 2020). The ability of *Brucella* to survive and replicate within host cells is a key pathogenic feature, as mutant strains that lack this capability are typically avirulent (Celli et al., 2003). *Brucella* mainly achieves immune evasion by manipulating monocytes/macrophages and neutrophils, utilizing virulence factors to regulate host signaling pathways, and employing a unique intracellular parasitic strategy.

### 2.1 *Brucella* manipulates neutrophils

Mora-Cartín et al. (2019), using a C57BL/6 mouse model, discovered that *B. abortus* 2308 (S2308) also achieves immune evasion by manipulating polymorphonuclear neutrophils (PMNs). PMNs are the most abundant effector cells in the innate immune system, rapidly migrating to infection sites to phagocytose *Brucella* during the early stages of infection. However, the bacteria exploits its lipopolysaccharide (LPS) to induce phosphatidylserine externalization on infected PMNs, generating an “eat me” signal, while concurrently suppressing the release of pro-inflammatory cytokines, such as interleukin (IL)-1β and tumor necrosis factor (TNF)-α, thereby dampening the classical inflammatory response (Mora-Cartín et al., 2019). When apoptotic PMNs carrying *Brucella* are cleared by macrophages or DCs via efferocytosis, the bacteria enter host cell phagosomes through this non-inflammatory pathway and subsequently escape to the endoplasmic reticulum (ER), where they establish a replicative niche. Macrophages infected via this route exhibit M2 polarization features (e.g., high IL-10 expression and low inducible nitric oxide synthase [iNOS] expression), which suppress Th1-type immune responses (e.g., reduced interferon [IFN]-γ secretion), thereby facilitating intracellular bacterial replication. Conversely, the specific antibody-mediated depletion of PMNs deprives *Brucella* of this crucial dissemination vehicle. This promotes M1 macrophage polarization (e.g., increased iNOS and IL-6 secretion) and enhances Th1-type immune responses (e.g., increased IFN-γ secretion), ultimately improving intracellular clearance of the pathogen (Mora-Cartín et al., 2019). However, Barquero-Calvo et al. (2007) chronically depleted polymorphonuclear neutrophils (PMNs) in Balb/c mice by administering the monoclonal antibody RB6. Their findings revealed that the absence of PMNs did not significantly alter the splenic colonization levels of S2308, suggesting that PMNs may play a limited role in the host’s defense against *Brucella* infection.

### 2.2 *Brucella v*irulence factors

#### 2.2.1 LPS

*Brucella* spp. are bacilli or coccobacilli that lack fimbriae, capsules, spores, cytolysins, extracellular enzymes, and exotoxins; their virulence is primarily manifested by invasion and proliferation (Bialer et al., 2020; Zheng et al., 2024; Lee et al., 2014; Głowacka et al., 2018). The lack of the aforementioned PAMPs further diminishes the detection of *Brucella* by pattern recognition receptors (PRRs) (Bialer et al., 2020). *Brucella* LPS has three main components: lipid A (the inner layer), an oligosaccharide core, and O antigen (the outer layer) (Yang X. et al., 2020; Bialer et al., 2020). The lipid A fatty acid chain (C28) of *Brucella* is longer than that (C12–C16) of *Escherichia coli*, and the LPS of smooth *Brucella* has a lower negative charge than that of rough *Brucella*. These properties decrease the activation of Toll-like receptor 4 (TLR4) by the LPS of *Brucella*, thus weakening the innate immune response of the hosts (Atluri et al., 2011; Smith et al., 2013). In addition, classification of *Brucella* into rough (*B. canis* and *B. ovis*) and smooth (*B. melitensis*, *B. suis*, and *B. abortus*) variants is based on reduced or absent O antigen of LPS in the rough variants (Fernandez-Prada et al., 2003; Bialer et al., 2020). The lack of a free hydroxyl group on the *Brucella* O antigen prevents it from binding to complement C3, thus suppressing the production of anaphylatoxins C3a and C5a (Barquero-Calvo et al., 2007; Atluri et al., 2011). Because C3a, C5a, and TLR 4 synergistically induce the production of pro-inflammatory cytokines, the characteristic LPS structure enables *Brucella* to evade the TLR4- and complement-mediated innate immune responses (Atluri et al., 2011). Studies have shown that the O antigen of LPS in smooth strains is a key factor for the entry of *Brucella* into mammalian cells (Martín-Martín et al., 2008). The structural features of the above-mentioned LPS (such as weak TLR4 activation and complement-binding inhibition) significantly reduce its immunogenicity, resulting in the host’s difficulty in generating protective immunity following *Brucella* infection or vaccination with an attenuated live vaccine.

#### 2.2.2 Flagella

Halling (1998) discovered that *B. abortus* possesses flagellar biosynthesis genes. Subsequent studies have demonstrated that most, if not all, *Brucella* strains have the genetic potential for flagella production but lack chemotaxis genes, precluding motility (Roop et al., 2021). *B. melitensis* 16M can synthesize sheathed flagella, but is non-motile because of the absence of chemotaxis genes (Fretin et al., 2005).

*In vitro* cell experiments have shown that *Brucella* flagellin lacks the key amino acid residues required to activate TLR5, preventing recognition by TLR5 and thus aiding in immune evasion. However, some studies indicate that *Brucella* flagellin eventually enters the cytoplasm of infected host cells, where it triggers an inflammasome-mediated inflammatory response, which is accompanied by the formation of granulomas. This process limits the extensive proliferation of *Brucella*, prevents excessive activation of the host immune response, and facilitates the establishment and maintenance of chronic infection in the host. Experimental evidence has demonstrated that flagellin-deficient *Brucella* mutants, compared to the wild-type strain, induce significantly higher bacterial burdens in the spleen and liver of infected mice, along with more severe pathological damage. The uncontrolled inflammatory damage induced by the flagellin-deficient strain hinders the establishment of chronic infection, further confirming that the presence of flagellin plays a positive role in the survival and infection process of *Brucella* (Terwagne et al., 2013).

#### 2.2.3 Type IV secretion system

A T4SS is crucial for *Brucella* trafficking in phagocytes and non-phagocytes. More specifically, T4SSs secrete effector proteins into host cells, manipulating intracellular endosome transport to avoid the lysosomal degradation and killing of *Brucella*, thus facilitating the formation of *Brucella*-containing vacuoles (BCVs) of the replicative type (rBCVs) within the cells. Furthermore, these effector proteins regulate immune responses, aiding in the proliferation and survival of *Brucella* within the body.

##### 2.2.3.1 Composition of the *Brucella* T4SS

The *Brucella* T4SS is a multiprotein complex that is regulated and expressed by the virB operon, comprising genes *virB1*–*virB12*. This T4SS mediates the replication, transport, and survival of *Brucella* within cells, while manipulating the innate and adaptive immunity of the hosts (Xiong et al., 2021; Zheng et al., 2024). The virB operon is found in all *Brucella* genera and is highly conserved (Xiong et al., 2021). The *Brucella* T4SS crosses the bacterial outer membrane and cell wall to secrete its effector proteins into host cells, altering normal intracellular transport processes or modifying the infection-triggered immune response (Myeni et al., 2013; Xiong et al., 2021; Spera et al., 2018; Zheng et al., 2024). Because *virB* mutants cannot replicate or survive within host cells and exhibit attenuated virulence in mouse models, it can be reasonably concluded that the T4SS is crucial for the replication, survival, and virulence of *Brucella* (Myeni et al., 2013). Although this T4SS is swiftly activated, peaking within 5 h post invasion, its activity is suppressed after the formation of rBCVs (Xiong et al., 2021).

**FIGURE 1:**
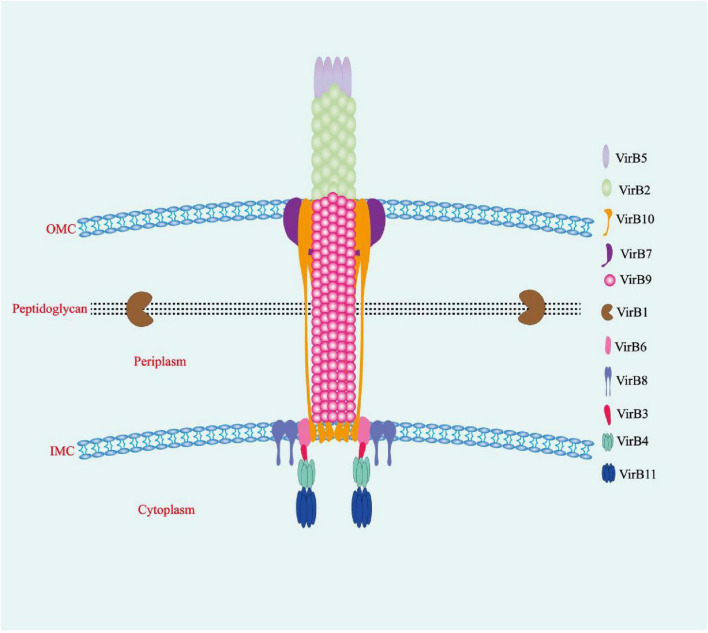
The T4SS of *Brucella*. The multiprotein T4SS complex, encoded by the virB operon, is involved in a series of intracellular activities and comprises three interconnected parts: the inner membrane complex (VirB3, VirB4, VirB6, and VirB8), the outer membrane core complex (VirB7, VirB9, and VirB10), and the external pilus (VirB2 and VirB5). OMC, outer membrane complex; IMC, inner membrane complex.

Each gene in the virB operon has a unique role, encoding proteins with distinct structures and functions. VirB proteins are functionally classified into four categories: ATPases, core components, surface-exposed components, and other components (Xiong et al., 2021). The ATPases include VirB4 and VirB11, which fuel T4SS assembly and effector protein transfer. However, the precise location of VirB11 at the bacterial inner membrane in relation to VirB4 is uncertain, and whether it aggregates below VirB4 or on the cytoplasmic side is also unclear (Bergé et al., 2017; Xiong et al., 2021). The core component is composed of VirB6–VirB10, which interconnect to create the T4SS translocation channel used to secrete effector proteins into host cells. VirB6 is an endosomal protein with a periplasmic N-terminal domain (NTD), five transmembrane domains, and a cytosolic C-terminus. The interaction of the VirB6 periplasmic NTD with VirB8 and VirB10 is crucial for the secretion of effector protein substrates via the endomembrane. VirB8 binds to VirB6 as a dimer and contains a periplasmic spatial domain, a single transmembrane helical structure, and a cytosolic C-terminus. VirB10 is a unique protein that spans the bacterial inner membrane to the outer membrane. Its N-terminus crosses the inner membrane, a central proline-rich region that traverses the periplasmic space, while its C-terminus forms the outer membrane pore. Furthermore, both the N-terminal and C-terminal domains of VirB10 are involved in interactions with other components of the T4SS. This structure enables VirB10 to play a key role in the T4SS, linking a wide range of proteins and aiding in their signaling interactions (Thanassi et al., 2012). VirB10 encapsulates VirB9, a periplasmic protein with an NTD and CTD that interacts with VirB7 to construct the inner wall of the T4SS core complex. VirB7, an N-terminally acetylated lipoprotein inserted in the outer membrane with its bulk extending into the periplasm, is vital for maintaining T4SS stability (Fronzes et al., 2009; Xiong et al., 2021). The surface-exposed component is composed of VirB2 and VirB5, which interact to form T-pili (Bergé et al., 2017). VirB2 is the major T-pilus subunit, creating columnar structures on the bacterial surface that facilitate effector protein transfer and convey target signal peptides across the inner membrane (Bergé et al., 2017; Keriel et al., 2015; Xiong et al., 2021). VirB5, a minor T-pilus subunit, sits at the apex, functioning as a specific adhesin that targets the host cell receptor (Aly and Baron, 2007; Deng H. et al., 2019; Xiong et al., 2021). Other components consist of VirB1 and VirB12. The NTD of VirB1 is a lytic transglycosylase that facilitates T4SS assembly by locally cleaving peptidoglycans (Ward et al., 2002; Xiong et al., 2021). The C-terminal fragment of the protein, known as VirB1*, can be cleaved and secreted outside of the bacterial cell, where it interacts with T-pilus subunits to enhance the assembly of T-pili (Zupan et al., 2007; Xiong et al., 2021). The precise location of VirB12 remains unknown, but it has been confirmed that VirB12 is a cell surface protein that triggers antibody responses in *Brucella*-infected animals. Overall, the T4SS comprises three parts: the inner membrane complex (IMC; VirB3, VirB4, VirB6, and VirB8), the outer membrane complex (OMC; VirB7, VirB9, and VirB10), and the external pilus (VirB2 and VirB5) (Bergé et al., 2017; Figure 1). VirB1, VirB7, and VirB12 are non-essential components of the T4SS, whereas VirB2–6 and VirB8–11 are essential. VirB1–11 exist in all *Brucella* species, whereas VirB12 is found only in *B. abortus*, *B. melitensis*, and *B. suis* (Xiong et al., 2021).

##### 2.2.3.2 T4SS-secreted effector proteins

The coexistence of *Brucella* with its hosts relies on its effector proteins, which are secreted via the T4SS and non-T4SSs. These effectors interact with a variety of molecules within host cells to regulate cellular physiology, altering the intracellular environment for the growth and proliferation of the bacteria and even facilitating bacterial invasion and overflow. The activities of effector proteins are precisely modulated through timed and spatial regulation of their synthesis and function (Ellis and Machner, 2024). Key effector proteins secreted by the *Brucella* VirB T4SS include RicA, VceC, VecA, BtpA (also known as Btp1 and TcpB), BtpB, BspA, BspB, BspF, BPE005, BPE123, BPE043, BPE275, SepA, BspC, BspE, BspL, NyxA, BspI, BspJ, NpeA, RS15060 and RS10635, which manipulate the signaling pathways of host cells to influence immune responses (Xiong et al., 2021; Smith et al., 2013; Kaplan-Türköz et al., 2013).

**FIGURE 2:**
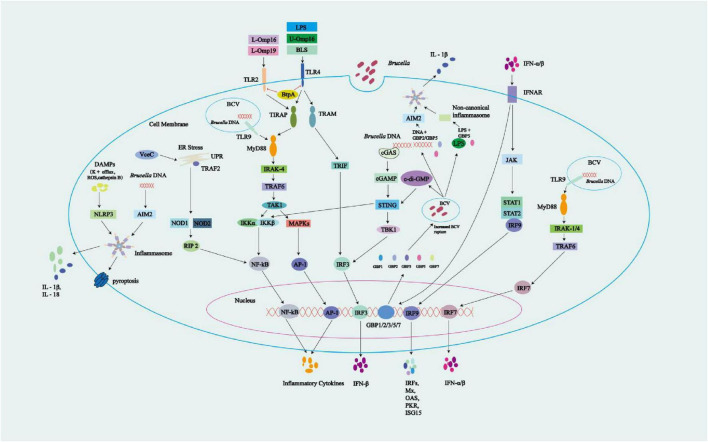
Overview of innate immune signals during *Brucella* infection. *Brucella*-associated molecular patterns can be detected by PRRs. TLR2 is activated by the lipidated *Brucella* OMPs L-Omp16 and L-Omp19, while TLR4 activation is induced by LPS, *Brucella* lumazine synthase (BLS), and unlipidated U-Omp16. TLR9 is activated by *Brucella* DNA. TLR activation triggers intracellular signaling via the MyD88-IRAK-4 and TRIF-TRAF3 pathways, leading to the activation of NF-κB, MAPK, IRF3, and IRF7, and the production of inflammatory cytokines and IFN-α/β. *Brucella*-derived BtpA/Btp1/TcpB may interfere with the TLR signaling pathways through its targeting of TLR2 and TLR4, impacting their downstream signaling processes. Cytoplasmic sensors also participate in the recognition of *Brucella* DNA, which activates cGAS. Additionally, STING responds to *Brucella*-released c-di-GMP, leading to IFN-β production via STING and IRF3 activation. Autocrine IFN-α/β engages IFNAR, triggering JAK-STAT1-IRF9 signaling, and upregulating ISG transcription to produce proteins including Mx, OAS, PKR, and ISG15. Activation of IFNAR significantly upregulates the expression of guanylate-binding proteins (GBPs) 1–3, 5, and 7. These GBPs promote the rupture of BCVs, leading to the release of components such as c-di-GMP, genomic DNA (gDNA), and LPS. The released gDNA activates the AIM2 inflammasome through the cooperative action of GBP5 and GBP2, while LPS triggers the non-canonical inflammasome pathway via GBP5. Together, these processes contribute to the production of proinflammatory cytokines such as IL-1β. Other cytoplasmic receptors can also be activated: (1) VceC, an effector protein produced by *Brucella*, activates the UPR, recruits TRAF2 onto the ER, and triggers the NOD1/2-RIP2-NF-κB pathway for cytokine production; (2) Absent in melanoma 2 (AIM2), which responds to *Brucella* gDNA; and (3) NLRP3, which is activated by *Brucella*-associated DAMPs (e.g., K^+^ efflux, ROS, and cathepsin B). Activation of the AIM2 and NLRP3 inflammasomes results in the release of pro-inflammatory cytokines, which may trigger pyroptosis. AIM2 and NLRP3 inflammasomes activation leads to caspase-1 activation, which simultaneously induces pyroptosis and promotes cytokine maturation.

In S2308, the effector proteins RicA and BspB play key roles in the intracellular trafficking of the bacteria within murine BMDMs. RicA binds to inactive Rab2 and promotes its recruitment to the BCV, disrupting vesicular transport and interfering with the host secretory pathway, leading to Golgi fragmentation. In the absence of BspB, RicA inhibits the formation of rBCV and bacterial replication. BspB, on the other hand, remodels Golgi-associated vesicular transport and compensates for the negative effects of RicA. These two proteins interact as described above, contributing to the biogenesis of rBCV, bacterial replication, and the regulation of Golgi-associated vesicular transport, thereby promoting bacterial proliferation (Smith et al., 2013). VceC targets the ER and binds to the glucose-regulated protein 78/binding immunoglobulin protein (GRP78/BiP), activating the inositol-requiring enzyme 1α (IRE1α) to elicit an unfolded protein response (UPR). Concurrently, upon activation, IRE1α facilitates the recruitment of TNF receptor-associated factor 2 (TRAF2) to the ER membrane. Together with nucleotide-binding oligomerization domain-containing proteins 1 and 2 (NOD1/2) and receptor-interacting protein kinases 2 (RIP2), this interaction leads to the induction of pro-inflammatory cytokines (e.g., IL-6) via the nuclear factor kappa-B (NF-κB) pathway, thereby initiating the inflammatory responses of the host. This inflammatory response occurs not only in mice but also in cells cultured *in vitro*, such as HeLa, HEK293, and BMDMs (de Jong et al., 2013; Keestra-Gounder et al., 2016; Figure 2). UPR represents a monitoring pathway that detects *Brucella* invasion of the ER, triggering inflammatory responses. Although the UPR-induced NF-κB signaling pathway may represent a novel host monitoring mechanism targeting the effector proteins of *B. abortus*, this pathogen appears to leverage this pathway to its advantage *in vivo*. Studies have shown that the VceC-deficient mutant strain exhibits a 50% reduction in colonization in the spleens of mice at 4–8 weeks compared to the wild-type strain, although there is no difference in initial colonization. This suggests that VceC provides a moderate advantage for long-term colonization (de Jong et al., 2013). Additionally, T4SS-mediated inflammation in mouse models can lead to abortion. Therefore, it may enhance *Brucella*’s survival adaptability, as abortion is a key route for the pathogen to spread in its natural bovine host (de Jong et al., 2013). In ovine trophoblast cells cultured *in vitro*, VceC secreted by *B. suis* S2 helps maintain GRP78/BiP expression, preserves ER homeostasis, and inhibits apoptosis by downregulating CHOP expression, thus promoting *Brucella* survival (Zhi et al., 2019). Research also indicates that the VceA-deficient strain can induce autophagy and inhibit apoptosis in human trophoblast cells (HPT-8), contributing to the persistence of *Brucella* in host cells (Zhang et al., 2019).

BtpA/Btp1/TcpB is present in *B. abortus* and *B. melitensis*, but absent in *B. suis* (Xiong et al., 2021). BtpA/Btp1/TcpB contains a Toll/IL-1 receptor (TIR) domain that competes with downstream signaling molecules of TLRs, inhibiting the TLR2 and TLR4 pathways. More specifically, this TIR domain mimics the structure of MyD88 adaptor-like (MAL) protein (also known as TIRAP). BtpA/Btp1/TcpB binds to the plasma membrane through its NTD to mimic the function of MAL, and then competes with MAL for binding to TLR4, thus interfering with the MyD88 pathway. This interaction inhibits the activation of NF-κB in host cell signaling, dampening inflammatory responses. Meanwhile, BtpA/Btp1/TcpB poly-ubiquitinates and degrades MAL. Additionally, BtpA/Btp1/TcpB interacts with MAL via the Box1 region to decrease the MAL phosphorylation level, thereby inhibiting TLR4/TLR2-mediated NF-κB activation and DC maturation (Gómez et al., 2020; Xiong et al., 2021; Atluri et al., 2011; Hu et al., 2023; Hashemifar et al., 2017; Zheng et al., 2024; Sengupta et al., 2010; Figure 2). Dimitrakopoulos et al. (2013) studied the infection process of *B. melitensis* using primary human monocytes. During the infection, the TLR2-p38 family mitogen-activated protein kinases (MAPKs) signaling pathway exhibited delayed activation, which may be caused by the interference of the *Brucella* protein BtpA/Btp1/TcpB with TIR-mediated signal transduction. This delayed activation creates favorable conditions for *Brucella* to hide within its replication niche. Early in the infection, IL-1β is already produced, and its production mechanism is independent of the TLR2-MAPK pathway. It may be mediated by inflammasome activation, triggered by unknown effector molecules secreted early during the cell-bacteria interaction. In the later stages of infection, the TLR2-MAPK pathway is activated, leading to the production of TNF-α and IL-6, which depend on p38 activation and are associated with ERK. The production of IL-10 is regulated by p38 and JNK (Figure 2). Despite the production of pro-inflammatory cytokines such as TNF-α, IL-6, and IL-1β during infection, the survival and replication of *Brucella* are not significantly affected. This is largely due to the delayed activation in the early stage of infection, which allows *Brucella* to form rBCVs. These rBCVs shield the bacteria from immune attack. Approximately 3 h after infection, *Brucella* becomes enclosed within rBCVs, rendering pro-inflammatory cytokines less effective. Moreover, the resulting inflammatory response not only fails to eliminate the bacteria but may also contribute to granulomatous disease, disrupting normal immune clearance and allowing *Brucella* to persist within the disordered immune environment. Studies have also shown that MAPK activation benefits *Brucella* survival and replication, while MAPK inhibition suppresses it. This is because MAPK activity is involved in multiple intracellular processes critical for *Brucella* survival, including early endosomal trafficking, phagosome acidification, signal transduction for T4SS induction, and regulation of the autophagy pathway.

BtpB also possesses a TIR domain with functions similar to BtpA/Btp1/TcpB. *In vitro*, BtpB was shown to suppress inflammatory responses by blocking transduction of the TLR2, TLR4, TLR5, and TLR9 signaling pathways, inhibiting the maturation of DCs and the activation of the NF-κB signaling pathway (Xiong et al., 2021). Inhibition of TLR-mediated innate immunity and blockage of DCs maturation lead to reduced secretion of pro-inflammatory cytokines and weakened antigen presentation, impairing the activation of adaptive immunity and thus favoring the survival of *Brucella*. The TIR domains of both BtpA/Btp1/TcpB and BtpB exhibit nicotinamide adenine dinucleotide (NAD^+^) hydrolase activity, which lowers total NAD levels to modulate the metabolism and signaling of host cells, including the downregulation of DCs activation during infection (Coronas-Serna et al., 2020). BspA, BspB, and BspF specifically inhibit protein secretion from the ER, fostering *Brucella* replication. Researchers found that when S2308 was used to infect HeLa, the effector protein BspA localized to the ER and disrupted the trafficking process from the ER to the Golgi apparatus (Myeni et al., 2013). By targeting the Golgi and the ERGIC, BspB interacts with conserved oligomeric Golgi (COG) to modify its function. BspB remodels the secretion/trafficking of ERGIC-Golgi, facilitating the retrograde transport of COG-dependent Golgi vesicles to BCVs, providing a Golgi membrane source that boosts rBCV production and *Brucella* replication (Miller et al., 2017). BspF interacts with Arf6 GTPase-activating protein 1 (ACAP1), leading to reduced Arf6 activity. This disruption impairs Arf6/Rab8a-regulated recycling endosome (RE) to trans-Golgi network (TGN) trafficking, thereby promoting the recruitment of TGN-derived vesicles to rBCVs and facilitating *Brucella* replication within rBCVs (Borghesan et al., 2021). In addition, due to its crotonyltransferase activity, BspF reduces the crotonylation of the host protein p53, resulting in decreased p53 expression. This suppresses host cell apoptosis and supports the long-term intracellular survival of *Brucella* (Lin et al., 2023).

Researchers, using the S2308 wild-type strain, its bpe123 deletion mutant, and a complemented strain in BMDMs, HeLa cells, and human THP-1-derived macrophage models, demonstrated that the effector protein BPE123 facilitates *Brucella* survival, replication, and completion of its intracellular life cycle. This is achieved through the direct interaction of BPE123 with host α-enolase (ENO-1), which enhances its catalytic activity. Consequently, key glycolytic enzymes, such as ENO-1 and GAPDH, are enriched within the BCV microenvironment, providing the bacterium with essential metabolic substrates and energy (Marchesini et al., 2016). When S2308 infected HeLa cells, the J774 A.1 macrophage-like cell line, and BMDMs, differences between the sepA deletion mutant and the wild-type strain were analyzed. The sepA mutant exhibited defects in early intracellular replication and showed reduced efficiency in excluding the lysosomal marker LAMP-1. These findings suggest that SepA may be involved in inhibiting the fusion of BCVs with lysosomes (Döhmer et al., 2014; Zheng et al., 2024). BspL delays the formation of autophagy-related BCVs (aBCVs), akin to autophagosomes, and extends the lifespan of rBCVs (Luizet et al., 2021). NyxA facilitates the formation of rBCVs (Louche et al., 2023). BPE275 may induce the formation of rBCVs (Hashemifar et al., 2017).

BspI is a T4SS effector protein of *Brucella*, with a GTPase-activating protein (GAP) domain at its C-terminus. During *Brucella* infection, the ER-localized effector VceC activates the kinase IRE1, inducing the secretion of proinflammatory cytokines such as IL-6 and TNF-α. BspI interacts with IRE1 and suppresses VceC-induced IRE1 kinase activity through its GAP domain, thereby reducing the production of proinflammatory cytokines. This regulatory effect is independent of its interaction with the small GTPase RAB1B. Moreover, the inhibition of NF-κB activation by BspI also depends on its GAP domain. Via this mechanism, BspI negatively regulates inflammation elicited by *Brucella* infection, thereby modulating the host immune response and defense against the pathogen (Li C. et al., 2022).

BspJ is a nuclear regulatory protein of *Brucella* that can enter the host cell nucleus and downregulate the expression of NME/NM23 nucleoside diphosphate kinase 2 (NME2) and creatine kinase B (CKB). BspJ interacts with both NME2 and CKB, which are associated with energy metabolism pathways. It is currently hypothesized that BspJ may inhibit macrophage apoptosis directly or indirectly through these interactions, thereby facilitating the intracellular lifecycle of *Brucella* during infection. However, this mechanism requires further investigation. BspJ may be secreted via a mixed transport system rather than relying solely on the T4SS (Ma et al., 2020). NpeA binds to the GTPase-binding domain (GBD) domain of neural Wiskott-Aldrich syndrome protein (N-WASP), a process that may activate N-WASP. Once activated, the VCA domain of N-WASP interacts with the actin-related protein Arp2/3 complex, leading to its activation. The activated Arp2/3 complex promotes actin polymerization, a process that contributes to the early stages of bacterial infection and plays a role in the formation of rBCVs (Giménez et al., 2024).

Cellular and murine studies have demonstrated that deletion of the *rs15060* gene reduces bacterial load, thereby impairing the ability of *Brucella* to establish chronic infection (Yin et al., 2024). Researchers have found that *rs15060* knockdown attenuates bacterial virulence, indicating that RS15060 contributes to the enhancement of *Brucella*’s pathogenicity. RS15060 is also essential for maintaining normal envelope morphology; its absence results in increased bacterial length, thickened cell walls, and altered morphology. Furthermore, RS15060 deficiency reduces cell wall permeability. In terms of LPS biosynthesis, RS15060 plays a suppressive role, and its deletion leads to increased LPS production. RS15060 also inhibits host proinflammatory responses; deletion of this protein enhances inflammatory cytokine production by the host (Yin et al., 2025). However, the specific mechanisms underlying these observations remain to be elucidated.

Studies on the *B. melitensis* M5-90 strain have shown that its secreted effector protein BspE is a nuclear regulatory factor. Experiments in the murine macrophage cell line RAW264.7 and Balb/c mouse models revealed that BspE not only activates the host type I interferon response and NF-κB signaling pathway but also specifically interacts with the host RNA-binding protein PCBP1. This BspE-PCBP1 interaction blocks p53 signaling, thereby inhibiting macrophage apoptosis and promoting *Brucella* survival within host macrophages. Moreover, BspE enhances bacterial replication *in vivo* and contributes to pathological damage in infected mice, including inflammatory cell infiltration, tissue edema, and structural destruction of the spleen and liver (He et al., 2025). When the S2308 strain infects the human hepatic stellate cell line LX-2, its secreted effector BPE005 induces HSC fibrosis via the Cyclic Adenosine Monophosphate (cAMP)/Protein Kinase A (PKA)–transforming growth factor (TGF)-β1 signaling pathway. This fibrosis not only causes liver tissue damage but also facilitates the formation of an immune-evading microenvironment, including granulomas, extracellular matrix barriers, and reduced immune cell recruitment. This conditions support the chronic infection and long-term persistence of *Brucella* in the host (Arriola Benitez et al., 2016). At present, the functions of BPE043, BspC, and RS10635 remain undefined (Table 1).

**TABLE 1:** *Brucella* T4SS effectors and their functions.

Factor	Type	Function	References
RicA	T4SS	Regulating vesicle trafficking through interaction with Rab2.	(Smith et al., 2020)
VceC	T4SS	• Binding to Bip/Grp78, it activates the UPR and induces pro-inflammatory responses. • Increasing the expression of Bip/Grp78 to maintain ER homeostasis. • Reducing CHOP expression to inhibit apoptosis.	(de Jong et al., 2013; Keestra-Gounder et al., 2016; Zhi et al., 2019)
VceA	T4SS	Inhibiting autophagy and promoting apoptosis.	(Zhang et al., 2019)
BtpA/Btp1/TcpB	T4SS	• TIR domain and interaction with MAL, it inhibits TLR pathways. • Regulates metabolic signaling by reducing host NAD+ levels through NAD+ hydrolase activity.	(Zheng et al., 2024; Sengupta et al., 2010; Coronas-Serna et al., 2020)
BtpB	T4SS	• TIR domain, inhibiting TLR pathways. • Regulates metabolic signaling by reducing host NAD+ levels through NAD+ hydrolase activity.	(Xiong et al., 2021; Coronas-Serna et al., 2020)
BspA	T4SS	Inhibiting the secretory pathway and vesicle transportation.	(Myeni et al., 2013)
BspB	T4SS	• Inhibiting the secretory. • Compensating for the negative effects of RicA. • Interacts with COG, regulating vesicle trafficking and promoting rBCV generation.	(Myeni et al., 2013; Smith et al., 2020; Miller et al., 2017)
BspC	T4SS	Unknown	
BspE	T4SS	• Promoting inflammation response. • Interacting with PCBP1, blocking the P53 pathway to inhibit apoptosis.	(He et al., 2025)
BspF	T4SS	• Inhibiting the secretory. • Interacting with ACAP1, regulating vesicle trafficking. • Reducing p53 crotonylation modification, inhibiting apoptosis.	(Myeni et al., 2013; Borghesan et al., 2021; Lin et al., 2023)
BPE123	T4SS	Interacting with ENO-1, utilizing the host’s glycolysis pathway to provide energy for Brucella.	(Marchesini et al., 2016)
BPE005	T4SS	Inhibiting cAMP/PKA pathway, relieving negative regulation on TGF-β1, promoting HSC fibrosis, supporting *Brucella* survival.	(Arriola Benitez et al., 2016)
BPE275	T4SS	Inducing rBCV formation.	(Hashemifar et al., 2017)
BPE043	T4SS	Unknown	
SepA	T4SS	Inhibiting BCV fusion with the lysosome.	(Döhmer et al., 2014)
BspL	T4SS	Delays aBCV formation.	(Luizet et al., 2021)
BspI	T4SS	Inhibiting pro-inflammatory cytokine release.	(Li C. et al., 2022)
BspJ	T4SS	Interacting with NME2 and CKB to inhibit cell apoptosis.	(Ma et al., 2020)
NyxA	T4SS	NyxA interacts with SENP3, indirectly promoting rBCV formation.	(Louche et al., 2023)
NpeA	T4SS	Promoting rBCV formation.	(Giménez et al., 2024)
Rs15060	T4SS	Maintaining membrane integrity and inhibiting inflammation.	(Yin et al., 2025)
Rs10635	T4SS	Unknown	
NyxB	Non-T4SS	NyxB interacts with SENP3, indirectly promoting rBCV formation.	(Louche et al., 2023)
PrpA	Non-T4SS	• Promoting B cell proliferation and antibody secretion by utilizing eukaryotic S-palmitoylation modification, and facilitates Brucella invasion of macrophages through ADCP. • Inhibiting inflammation. • Mediates the host’s early transient non-responsive state.	(Spera et al., 2018; Spera et al., 2014; Spera et al., 2006)
Cu-Zn SOD	Non-T4SS	Inactivating Sar1, inhibiting *Brucella* growth.	(Liu et al., 2018)

#### 2.2.4 Non-T4SS-secreted effector proteins

*Brucella* effector NyxB facilitates rBCV formation, but the entry route into host cells remains unclear (Louche et al., 2023). Experiments using S2308 in BALB/c mice, HEK293, and J774 A.1 cell models have revealed that the effector protein PrpA acts as a mitogen for B cells and undergoes S-palmitoylation. This finding indicates that *Brucella* hijacks eukaryotic post-translational modification machinery to enhance its own intracellular survival and replication (Spera et al., 2018). S-palmitoylated PrpA migrates to the plasma membrane of the murine macrophage cell line J774A.1 and binds to surface receptors, triggering these macrophages to release soluble factors that promote B lymphocyte proliferation. This elevates the number of B cells and thus the titers of specific immunoglobulin (Ig) antibodies that facilitate *Brucella* invasion into macrophages via antibody-dependent cellular phagocytosis (ADCP), thereby leveraging host humoral immunity for S2308 infection. Furthermore, opsonogenic antibodies against pathogens and their LPS can also facilitate infections *in vitro* (Spera et al., 2014; Spera et al., 2018). PrpA is also associated with the downregulation of IFN-γ and TNF-α, along with the upregulation of TGF-β1 in mice, thereby turning the protective pro-inflammatory reaction into an anti-inflammatory reaction (Spera et al., 2014; Spera et al., 2006). The splenocytes of *B. abortus*-infected mice exhibited early, transient non-responsiveness to *E. coli* LPS and concanavalin A, but this phenomenon was absent under infection with *B. abortus* carrying a *prpA* mutation. Additionally, the *prpA* mutant strain had a diminished capacity for chronic infection in mice. These findings indicate that the early, transient non-responsiveness of the host immune system mediated by PrpA is essential for the establishment of chronic *Brucella* infection (Spera et al., 2006). *Brucella* Cu-Zn superoxide dismutase (Cu-Zn SOD) is a periplasmic protein. Studies have shown that immunization with recombinant Cu-Zn SOD protein can protect mice from infection with S2308. In addition, Cu-Zn SOD can be translocated into host cells independently of the T4SS. Within host cells, Cu-Zn SOD inhibits intracellular bacterial growth by inactivating Secretion-Associated Ras-related protein 1 (Sar1). Notably, this inhibitory effect is independent of reactive oxygen species (ROS) and nitric oxide (NO) production (Liu et al., 2018).

#### 2.2.5 Outer membrane proteins

OMPs play multifaceted roles in the viability of *Brucella*. Structurally, OMPs are associated with the outer membrane, which is tightly linked to the peptidoglycan layer in the cell wall (Tibor et al., 1999). Together with the LPS O antigen (O-polysaccharide chains), OMPs defend *Brucella* against the complement system and antimicrobial peptides (Roop et al., 2021). OMPs also facilitate direct *Brucella*–host cell interaction during invasion, aiding in the entry of rough strains into host cells. For example, Omp25d and Omp22 play important roles during the entry of *B. ovis* into mammalian cells. In smooth *Brucella* strains, there is no evidence indicating that OMPs perform such functions, as the O antigen mask surface antigens like OMPs, thereby hindering their interaction with host cells (Martín-Martín et al., 2008). Finally, OMPs are key virulence factors for the intracellular immune evasion and survival of *Brucella*.

The common OMPs can be classified by molecular weight, structure, and function as follows: (1) outer membrane lipoproteins, including Omp10 (10 kDa), Omp16 (16 kDa), and Omp19 (19 kDa) (Tibor et al., 1999); (2) outer membrane porins, including Omp2a and Omp2b (36–38 kDa) (Cloeckaert et al., 1996a); (3) virulence-associated OMPs, including Omp22 (also known as Omp3b), Omp25 (also known as Omp3a), Omp25b, Omp25c, Omp25d, and Omp28 (also known as Bp26) (25–28 kDa) (Cloeckaert et al., 1996a; Cloeckaert et al., 1996b; Guzman-Verri et al., 2002); and (4) other OMPs, including Omp31 and Omp31b (31–34 kDa) (Cloeckaert et al., 1996a). The following section focuses on the extensively studied outer membrane proteins Omp25, Omp31, Omp16, and Omp19.

##### 2.2.5.1 The Omp25/Omp31 family

The Omp25/Omp31 family comprises seven homologous OMPs: Omp31, Omp31b, Omp25, Omp25b, Omp25c, Omp25d, and Omp22. Research on six classical *Brucella* species (*B. melitensis*, *B. abortus*, *B. suis*, *B. ovis*, *B. canis*, and *B. neotomae*) revealed varying expression profiles of these proteins, with no single species producing all seven homologous OMPs (Martín-Martín et al., 2009).

###### 2.2.5.1.1 Omp25

Omp25 is a transmembrane protein highly conserved among *Brucella* species. In addition to being a major component of the *Brucella* outer membrane, Omp25 exhibits antigenicity in both murine and cattle. Immunization of mice with *Brucella* membrane extracts rich in Omp25 or with native Omp25 formulated with oil adjuvant confers significant protection against challenge with virulent strains (Dubray and Bézard, 1980; Montaraz and Winter, 1986; Winter and Rowe, 1988; Edmonds et al., 2002). Researchers constructed omp25 deletion mutants of *B. abortus*, *B. melitensis*, and *B. ovis*, designated BA25, BM25, and BO25, respectively. In mouse infection experiments, significantly reduced colony-forming units (CFUs) in the spleens were observed at 18 weeks post-infection with BA25 and at 4 weeks with BM25 compared to their virulent parental strains. Notably, in mice infected with BO25, splenic CFUs were markedly reduced throughout weeks 1–8, and the mutant strain was completely cleared by week 8. These findings indicate that the virulence of BA25, BM25, and BO25 is attenuated, resulting in impaired intracellular survival of *Brucella* in host cells. The attenuation of virulence in BO25 was more pronounced than that observed in BA25 and BM25. This may be attributed to the fact that *B. ovis*, the natural host strain, does not express O antigen. Therefore, the deletion of Omp25 likely had a greater impact on the stability of the bacterial outer membrane in *B. ovis*, resulting in a marked attenuation of virulence (Edmonds et al., 2002). Collectively, these findings suggest that Omp25 plays a critical role in the virulence of *Brucella*.

To further investigate the regulatory mechanisms underlying the reduced virulence caused by the absence of the Omp25 gene, Zhu et al. (2018) constructed an omp25-deficient mutant strain of *Brucella* melitensis (M5-90-Δomp25) and infected RAW264.7 macrophages with it. They found that infection with M5-90-Δ*omp2*5 altered the intracellular expression of miRNAs, with certain miRNAs (mmu-miR-146a-5p, mmu-miR-155-5p) being upregulated, while others (mmu-miR-149-3p, mmu-miR-5126) were downregulated. In addition, the researchers conducted mRNA expression profiling of the infected cells, identifying 967 differentially expressed genes (DEGs). They then used software tools such as TargetScan, miRanda, and PicTar to predict the potential target genes of these differentially expressed miRNAs. The results indicated that 17 genes might be potential targets of mmu-miR-149-3p, with Tbr1 being simultaneously targeted by mmu-miR-5126. qRT-PCR analysis confirmed that the expression of 9 predicted target genes was upregulated. Further studies confirmed that *Bcl6b*, *Nos2*, *Ikbke*, *Slc31a2*, *Dusp16*, *Ifit1*, *Slc7a11*, *Il1rl1*, and *Olr1* are the target genes of mmu-miR-149-3p. In humans, *BCL6b* is expressed in a small subset of antigen-stimulated CD8^+^ T cells and plays a key role in enhancing the secondary response of memory CD8^+^ T cells. *NOS2* is involved in the generation of NO, which serves as a cellular defense mechanism against *Brucella* infection. Dual-specificity phosphatases (DUSPs) belong to the protein phosphatase family. In macrophages, the RNA expression of Dusp16 can be induced by TLR stimulation. Dusp16 preferentially dephosphorylates c-Jun N-terminal kinase and MAPKs. When macrophages are infected with M. tuberculosis, the Eis (enhanced intracellular survival protein) produced by M. tuberculosis N^2^-acetylates Lys55 of DUSP16, thus initiating a suppression of autophagy and phagosome maturation, inhibiting the host immune response and aiding the survival of Mycobacterium tuberculosis within the cells. Interferon-induced proteins (IFITs) are important mediators of innate antiviral immunity in mammals, and IFIT1 has been shown to be an effective inhibitor of alphavirus replication. In a mouse model, intraperitoneal injection of LPS induces upregulation of Olr1 expression in the lungs, indicating that Olr1 is involved in LPS-induced pulmonary inflammation. In summary, the upregulation of these genes in the infection models suggests that the functional mechanism of Omp25 is complex, potentially involving multiple signaling pathways and numerous signaling molecules.

Degos et al. (2020) further elucidated the pivotal role of Omp25 in immune evasion mechanisms. The *B. abortus* outer membrane protein Omp25 interacts with SLAMF1 (also known as CD150), a member of the signaling lymphocyte activation molecule (SLAM) family. This interaction inhibits the nuclear translocation of NF-κB in infected bone marrow-derived dendritic cells (BMDCs), subsequently leading to decreased expression of co-stimulatory molecules on the DC surface, reduced cytokine secretion, and ultimately, suppression of the inflammatory response. Although the Omp25/SLAMF1 axis has no apparent effect on *Brucella* replication in the acute infection stage, it does promote the persistence of *Brucella* in the chronic stage, potentially aiding immune evasion and the establishment of chronic infection.

Jubier-Maurin et al. (2001), in their studies on *B. suis*, further elucidated the Omp25-mediated regulation of host immune responses from an additional perspective. They demonstrated that Omp25 from this bacterium inhibits TNF-α production during the infection of human THP-1 macrophages. In humans, such an inhibitory effect may compromise host defense through several mechanisms. First, it could attenuate innate immunity. Second, it could reduce the production of IL-12, thereby suppressing the helper T cell (Th)1 immune response and skewing it toward a Th2 response, a typical manifestation of chronic brucellosis in humans featuring high antibody titers and a weak delayed-type hypersensitivity reaction. Additionally, high levels of murine monoclonal antibodies against Omp25 can block the immunosuppressive effects of Omp25, increasing TNF-α production and thereby contributing to protective immunity against *Brucella* species (Jubier-Maurin et al., 2001). This effect may be more important in rough strains than in smooth strains, because the LPS O antigen in smooth strains creates steric hindrance, whereas Omp25 is more readily recognized by antibodies in rough strains (Jubier-Maurin et al., 2001).

###### 2.2.5.1.2 OMP31

Omp31 plays a crucial role in the infection mechanism of *Brucella*. L. Verdiguel-Fernández et al. (2017) constructed an Omp31 deletion mutant (LVM31) of *B. melitensis*. The study found that the absence of Omp31 disrupts the integrity of the outer membrane, leading to a significant decrease in the bacterium’s internalization ability, intracellular survival, and replication capacity, thereby reducing virulence. Additionally, Omp31 also has the ability to bind hemoglobin. Iron is essential for *Brucella* survival, with macrophage-derived heme as its source. Omp31 is a heme-binding protein (HBP) that sequesters iron from heme. In addition, Omp31 expression is iron-regulated, increasing under iron deficiency. *B. abortus* lacks Omp31, but may utilize alternative heme uptake pathways, suggesting diverse iron acquisition strategies in *Brucella* (Delpino et al., 2006). Research has shown that Omp31 from *B. melitensis* 16M strain can inhibit apoptosis in RAW264.7 macrophages, which contributes to the long-term survival and replication of the bacteria within the host (Zhang K. et al., 2016). Additionally, in a central nervous system infection model, Omp31 has been found to induce autophagy in BV-2 microglial cells, thereby inhibiting the NF-κB p65 signaling pathway and regulating both the inflammatory response and the autophagic process (Wang et al., 2021a). Further details about Omp31, apoptosis, and autophagy will be discussed in Section 5.1, “Selective subversion of autophagy pathways,” and Section 5.4, “Apoptosis pathways.”

##### 2.2.5.2 Omp16, and Omp19

Lipoproteins Omp16, and Omp19 are expressed across all six classical *Brucella* species and biotypes (Tibor et al., 1999).

LPS was previously thought to be a key factor in inflammation caused by Gram-negative bacteria. However, the LPS of *Brucella* is structurally and functionally distinct from that of classical *Enterobacter* species, exhibiting a biological induction of pro-inflammatory mediators that is at least three orders of magnitude lower. Only high concentrations of *Brucella* LPS induce minimal cytokine production, which may be unimportant or have no substantial impact on the host inflammatory response. In addition, *Brucella* lipoproteins are ligands for TLR2 but not for TLR4, with immune effects that are lipid mediated and capable of inducing inflammatory cytokine production. Research on lipidated (L-) and unlipidated (U-) Omp16 and Omp19 revealed that L-Omp16 and L-Omp19 induce the secretion of a variety of cytokines (e.g., pro-inflammatory TNF-α, IL-6, and IL-12, and anti-inflammatory IL-10) by human monocytic THP-1 cells in a time- and dose-dependent manner (Figure 2). In contrast, both U-Omp16 and U-Omp19 failed to induce cytokine secretion by THP-1 cells at any tested concentration. Therefore, the N-terminal lipid-modified tripalmitoyl-Cys moiety of OMPs appears to be crucial for cytokine induction in monocytes/macrophages. This finding underpins the role of bacterial lipoproteins in inflammation, highlighting their specific interactions with TLR2 and the pivotal role of lipid modification in cytokine induction (Giambartolomei et al., 2004; Barrionuevo et al., 2008). L-Omp19 can induce IL-6 production via TLR2 in THP-1 cells. This IL-6 inhibits the IFN-γ-induced expression and nuclear translocation of IFN regulatory factor 1 (IRF-1), which suppresses transcription of the human leukocyte antigen complex (HLA) DR isotype and the class II transactivator (CIITA), thereby attenuating major histocompatibility complex II expression and antigen processing/presentation, aiding the immune evasion of *Brucella* (Barrionuevo et al., 2008; Velásquez et al., 2017). L-Omp19 can also stimulate human monocytes/macrophages via TLR2 to inhibit IFN-γ-induced FcγRI (CD64) expression and FcγRI-mediated phagocytosis (Barrionuevo et al., 2011). Furthermore, Omp19 acts as a protease inhibitor and plays a key role during the oral infection process of *Brucella*. It effectively defends against proteases in the intestine and lysosomal proteases in host cells (Pasquevich et al., 2019). Meanwhile, Omp19 works in synergy with urease secreted by *Brucella* (which helps the bacteria withstand the low pH environment of the stomach) and choloylglycine hydrolase (CGH, which grants the bacteria resistance to bile acids), collectively promoting *Brucella*’s ability to induce chronic infection through the oral route (Sangari et al., 2007; Delpino et al., 2007).

#### 2.2.6 Phosphatidylcholine

Phosphatidylcholine (PC) is commonly found in eukaryotes but is rare in prokaryotes, with only about 10% of bacteria containing it, mostly among those interacting with eukaryotic organisms. The cell membrane of *Brucella* is rich in PC (Aktas et al., 2010). *Brucella* may utilize PC to mimic eukaryotic features, thereby evading the host’s innate immune response and facilitating the establishment of long-term infection within the host. In bacteria, PC synthesis occurs via two pathways: phospholipid N-methylation (Pmt) and phosphatidylcholine synthase (Pcs). The Pmt pathway involves the gradual methylation of phosphatidylethanolamine (PE) into PC by the enzyme phospholipid N-methyltransferase (PmtA) using S-adenosylmethionine (SAM) as the methyl donor. In the Pcs pathway, Pcs catalyzes the condensation of choline with CDP-diacylglycerol to produce PC, a synthesis for which choline serves as an essential substrate (Aragón-Aranda et al., 2021). In *Brucella*, a mutant strain of *Brucella* abortus lacking PC in the outer membrane exhibits reduced virulence in mouse models (Roop et al., 2009). The PC/PE ratio in the outer membrane of *Brucella* affects its resistance to antimicrobial peptides and complement-mediated killing, and PC may also play a role in modulating the host’s response to infection (Roop et al., 2021). The Pcs pathway is unique to bacteria for PC synthesis, while the Pmt pathway is present in both certain eukaryotes and bacteria, with significant sequence differences between the Pmt enzymes involved in bacteria and eukaryotes. In eukaryotes, PC is primarily synthesized via the CDP-choline pathway, and they do not possess the Pcs pathway. Based on these differences, the development of novel antimicrobial strategies targeting the Pmt and Pcs enzymes of *Brucella* is considered feasible (Aktas et al., 2010).

Lee et al. (2014) identified that betaine aldehyde dehydrogenase (BADH) is another important virulence factor in *Brucella*. Choline is converted into betaine aldehyde under the action of choline dehydrogenase, and the enzyme encoded by the *BetB* gene, BADH, catalyzes the conversion of betaine aldehyde into glycine betaine (Velasco-García et al., 2006; Lee et al., 2014). Glycine betaine, recognized as one of the most effective osmoprotectants in both prokaryotic and eukaryotic organisms, enhances the osmotic stress resistance of *Brucella*, thereby facilitating its adaptation to the harsh intracellular environment within host cells. The presence of the *BetB* gene decreases the adhesion and entry of *Brucella* into host cells, possibly through expression of surface proteins and/or changes in the synthesis of OMPs and PC. *BetB* enhances BCV maturation, which boosts *Brucella* intracellular replication. Concurrently, compared with a *BetB*-deleted mutant, *BetB*-containing *B. abortus* exhibits reduced MAPK activation in phagocytes, thereby suppressing the normal immune response and activation of host cells. Choline metabolism is dependent on glycine betaine, and choline also participates in the synthesis of PC. Researchers speculate that the BADH in *Brucella* may influence the synthesis of PC by regulating choline and glycine betaine metabolism, thereby indirectly participating in the bacterium’s intracellular transport and survival processes (Lee et al., 2014).

## 3 Intracellular survival of *Brucella*

*Brucella* infection of host cells involves several steps, including adhesion, internalization, intracellular replication/survival, and transport (Yu et al., 2024). The adhesins of *Brucella* facilitate the binding of *Brucella* to host cells and/or extracellular matrix (ECM). These adhesins include the following: sialic acid-binding proteins SP29 and SP41, for binding to erythrocytes and epithelial cells; BigA and BigB proteins, which contain Ig-like domains for binding to adhesion molecules in epithelial cells; monomeric autotransporters BmaA, BmaB, and BmaC, for binding to ECM components, epithelial cells, osteoblasts, synovial cells, and trophoblasts; trimeric autotransporters BtaE and BtaF, for binding to ECM components and epithelial cells; Bp26, for binding to ECM components; and the T4SS protein VirB5 (Bialer et al., 2020; Xiong et al., 2021).

Adherence of *Brucella* to host cells occurs through a variety of receptors on the surface of those cells, resulting in ligand–receptor binding. *Brucella* enters host cells via either opsonic or non-opsonic pathways. Early in infection, prior to antibody production, *Brucella* invades cells via non-opsonic mechanisms. Specifically, the class A scavenger receptor (SR-A) on host cells binds to lipid A of the bacterial LPS, enabling the entry of *Brucella* via clathrin- and dynamin-dependent endocytosis pathways, with the involvement of membrane-bound lipid rafts, actin, and the cytoskeletal regulator Rho small GTPase. This route is applicable throughout infection, from early to late stages (Lee et al., 2013; Jimenez de Bagues et al., 2005; Guzmán-Verri et al., 2001; Atluri et al., 2011; Oliveira et al., 2008; Kim et al., 2004). However, as antibody levels increase during the intermediate and late stages of infection, *Brucella* also enters host cells via opsonization, wherein Fc receptors (FcRs) expressed on the phagocyte surface bind to antibodies, thereby facilitating bacterial entry (Atluri et al., 2011). Non-phagocytes have low susceptibility to *Brucella* uptake and weaker phagocytic ability (Chaves-Olarte et al., 2002). Professional phagocytes possess Fc and complement receptors, exhibit very high phagocytic efficiency. Research has shown that in the process of *Brucella* infection in phagocytes, using immune serum containing antibodies specific to *Brucella* to opsonize *B. suis* significantly enhances the phagocytosis process. Although the initial bacterial load inside the cells is higher after opsonization compared to untreated cells, bacterial proliferation within the cells is markedly reduced in opsonized infections. the Fc receptor (FcR) is associated with MAPKs. During opsonized *Brucella* infection, the FcR binds to its ligands, possibly influencing post-transcriptional expression of iNOS. The NO produced by iNOS can participate in bacterial killing. This suggests that opsonization, via receptors on macrophage surfaces, promotes bacterial phagocytosis and aids in bacterial destruction within the cells (Gross et al., 1998).

The entry of *Brucella* into host cells (phagocytes or non-phagocytics) triggers the formation of BCVs (Atluri et al., 2011). In BMDMs, HeLa cells, and NIH3T3 cells, BCVs interact with early endosomes (EE) in the cytoplasm to obtain early endosomal markers such as Rab5 and EEA-1, forming early endosomal BCVs (eBCVs) (Celli, 2019; Celli et al., 2003; Comerci et al., 2001; Chaves-Olarte et al., 2002). In HeLa and NIH3T3 cells, after *Brucella* infection, the EE containing the bacteria avoids fusion with late endosomes (LE) and lysosomes. The bacteria then alter their transport route and reach the ER through autophagosomes, forming rBCV (Chaves-Olarte et al., 2002; Comerci et al., 2001; Pizarro-Cerdá et al., 1998a; Pizarro-Cerdá et al., 1998b). In BMDMs, early eBCVs interact and partially fuse with LE and lysosomes. During this process, the BCV undergoes acidification, with a pH reaching 4.5–5.0, and acquires markers of LE and lysosomes, such as Rab7, LAMP-1, CD63, and RILP. Additionally, during this process, *Brucella* is exposed to proteases, ROS, reactive nitrogen species (RNS), and low pH conditions. Under these circumstances, 90% of *Brucella* will be eliminated by the lysosome (Starr et al., 2008; Celli et al., 2003; Gómez et al., 2020).

The formation of early and late eBCVs occurs within the first 8 h post infection (Celli, 2019). The acidic endosomal/lysosomal environment triggers T4SS expression, regulated by various factors at the transcriptional level. These regulatory factors include the vacuolar hijacking *Brucella* regulator (VjbR), *Brucella* luxR-like regulator (BlxR), and two-component regulatory system BvrR/BvrS, in addition to host-encoded histidine utilization regulator (HutC), RelA/SpoT homolog (Rsh), MarR-like sodium deoxycholate-responsive activator (MdrA), *Brucella* quorum-sensing regulator (BabR), and integration host factor (IHF) (Ke et al., 2015). The T4SS secretes various effector proteins that enable *Brucella* to escape from phagolysosomes and mediate the exclusion of phagolysosomal markers (e.g., LAMP-1) from the eBCV, resulting in the survival of approximately 10% of *Brucella* (de Jong et al., 2013; Zheng et al., 2024; Gómez et al., 2020). Furthermore, it has been observed in both phagocytic cells (e.g., mouse-derived macrophages) and non-phagocytic cells (e.g., mouse embryonic fibroblasts) that these effector proteins induce the UPR in host cells, activating the RNase of IRE1α and degrading *Blocis1* mRNA, resulting in decreased BLOCIS protein production (a process known as regulated IRE1-dependent mRNA decay). BLOCIS is a component of the BLOC-one-related complex (BORC), which is involved in the biosynthesis of lysosome-associated organelles and is reduced through *Brucella*-mediated downregulation of *Blocis1* expression. BORC also mediates the transport of late eBCVs to lysosomes, thus allowing *Brucella* to inhibit the fusion between late eBCVs and lysosomes (Wells et al., 2022; Celli et al., 2003). Meanwhile, in HeLa cells and macrophages, *Brucella*-secreted cyclic β-1,2-glucans (CβGs) disrupt the cholesterol-rich lipid rafts on the BCV membrane, impairing BCV maturation and thus preventing BCV fusion with lysosomes (Guo et al., 2023). T4SS-secreted effector proteins mediate the interaction of eBCVs containing viable *Brucella* with ER exit sites (ERES) and coat protein complex II (COPII) vesicles, a process regulated by the small GTPase Sar1, facilitating the fusion of eBCVs with the ribosome-associated ER membrane to acquire ER markers, including glucose-6-phosphatase, calnexin, calreticulin, Sec61, and PDI (Celli, 2019; Keriel et al., 2015; Starr et al., 2012; Guo et al., 2023; Myeni et al., 2013). During this process, the BCV recruits the small GTPase Rab2 and GAPDH, which are involved in regulating vesicle transport between the ER and the ERGIC. These factors contribute to the formation of the bacterial replicative niche and are essential for *Brucella* replication (Fugier et al., 2009).

**FIGURE 3:**
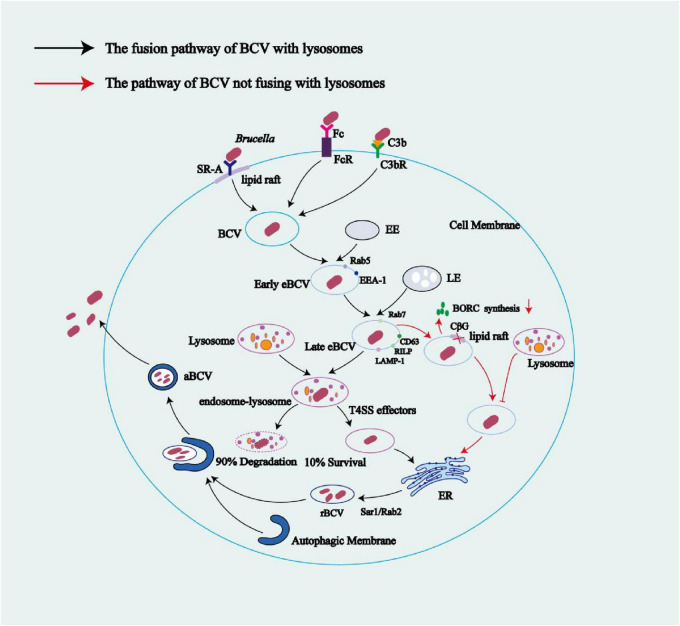
The life cycle of *Brucella* within macrophage cells. After binding to SR-A or FcR and C3bR receptors in lipid rafts on the surface of macrophage membranes, *Brucella* enters cells via endocytosis, forming BCVs. These BCVs interact with early endosomes to obtain the host Rab5 protein, forming early eBCVs. As early eBCVs mature, they gradually lose early endosomal markers (e.g., Rab5) and acquire characteristic markers of late endosomes (e.g., Rab7, CD63, RILP, and LAMP-1) forming late eBCVs. The fate of late eBCVs involves two distinct mechanisms: (1) fusion of late eBCVs with lysosomes, leading to clearance of 90% of the bacteria by lysosomal enzymes and the escape of the remaining 10% of viable bacteria through a regulatory mechanism, allowing them to reach the ER; and (2) *Brucella*-mediated inhibition of late eBCV–lysosome fusion through reduced BORC activity via bacterial replication. Meanwhile, *Brucella*-secreted CβGs suppress BCV maturation by disrupting the cholesterol-rich lipid rafts on the BCV membrane, thus blocking the fusion of eBCVs with lysosomes. Mediated by host factors such as Sar1 and Rab2, BCVs that reach the ER via any of the above pathways ultimately fuse with the ER membranes to form rBCVs. Active proliferation of *Brucella* inside rBCVs converts the rBCVs into aBCVs filled with numerous pathogenic cells. Later in the infection, aBCVs fuse with MVBs to form amphisomes, which then fuse with the plasma membrane for bacterial budding, which concludes the intracellular infection cycle. Black arrows indicate the fusion pathway of BCVs with lysosomes; red arrows indicate the pathway of BCV that does not involve lysosome fusion.

The rBCVs form at 8–12 h post infection, losing the lysosomal component LAMP-1. Host proteins including the small GTPase IRE1α, Yip1A, Atg9, and WD-repeat domain phosphoinositide-interacting protein 1 (WIPI1), as well as the COG complex, are crucial for rBCV biogenesis (Celli, 2019; Keriel et al., 2015; Starr et al., 2012; Guo et al., 2023). *Brucella* cells in rBCVs proliferate in large numbers from 12 to 48 h post infection (Celli, 2019). Additional studies have shown that the endosomal/lysosomal vesicles fuse with ER-derived autophagic vesicles, leading to the production of rBCVs (as detailed in 5.2 the ER stress pathways subsection below) (Taguchi et al., 2015). In BMDMs from C57BL/6J mice and in HeLa cells, during 48 to 72 h post-infection, the intracellular rBCVs become enveloped by crescent-shaped, autophagosome-like membrane structures, gradually developing into aBCVs. The formation of aBCVs requires the functioning of the classical autophagosome nucleation complex, bypassing that of the elongation complex, and requires the autophagy initiators beclin1, ULK1, Atg14L, and PI3Ks, but not the autophagy elongation proteins ATG5, ATG16L1, ATG4B, ATG7, and LC3B (Starr et al., 2012). Therefore, aBCV formation relies on the molecular mechanisms of autophagy initiation in the host. The fusion of aBCVs and multivesicular bodies (MVBs) forms amphisomes, which then fuse with the plasma membrane for bacterial budding, completing the intracellular *Brucella* cycle and initiating new infections (Spera et al., 2023). Smith et al. (2016) demonstrated that, in BMDMs, the formation of aBCVs and subsequent bacterial release also rely on the T4SS. In BMDMs and HeLa cells, before aBCVs form, the *Brucella*-secreted T4SS effector protein BspL engages with the homocysteine-inducible ER stress protein (Herp), a core component of ER-associated degradation (ERAD), stabilizing Herp and preventing its degradation. Once Herp has been hijacked and stabilized by BspL, Beclin-1 activity declines, which slows the formation of aBCVs, the final step of the intracellular cycle. This ultimately gives *Brucella* more time to proliferate in rBCVs (Luizet et al., 2021). The intracellular survival mode of *Brucella* limits its exposure to and activation of the innate immune system. Furthermore, because innate immunity is a bridge to adaptive immunity, the latter is also not effectively activated. Additionally, this survival mode undermines the efficacy of some antibiotics, favoring the intracellular parasitism of *Brucella* (Wells et al., 2022; Figure 3).

## 4 Common *Brucella* PAMPs and corresponding host PRRs

PRRs encompass a variety of components, including TLRs, cyclic GMP-AMP synthase (cGAS), absent in melanoma 2 (AIM2), and nucleotide-binding domain leucine-rich repeats (NLRs). TLRs located on cellular and endosomal membranes detect PAMPs from outside cells and inside endosomal lumens, respectively. In the cytoplasm, cGAS and AIM2 sense cytoplasmic double-stranded DNA. NLRs are also located in the cytoplasm, where they recognize PAMPs and damage-associated molecular patterns (DAMPs) (Kumar et al., 2011; Foley et al., 2015; Bai and Liu, 2019; Tupik et al., 2020). Upon activation of TLR2 by lipoproteins from *B. abortus*, such as lipidated outer membrane proteins L-Omp16 and L-Omp19, the adaptor protein TIRAP is recruited. TIRAP then binds to MyD88, which subsequently engages IRAK-4 through its death domain. Activated IRAK-4 promotes the oligomerization of TRAF6, leading to the activation of the key signaling molecule TAK1. TAK1 transmits signals through two major pathways: first, it activates the NF-κB pathway by phosphorylating the IKK complex (IKKα/β subunits), which then phosphorylates IκB. This phosphorylation targets IκB for ubiquitination and degradation, thereby releasing the NF-κB to translocate into the nucleus and promote transcription of pro-inflammatory cytokines such as TNF-α and IL-6. Second, TAK1 activates the MAPK pathway, which leads to the activation of AP-1 transcription factors that regulate genes involved in inflammation. TLR4 is activated upon recognition of *B. abortus* LPS, *Brucella* lumazine synthase (BLS), and U-Omp16. It signals through two distinct pathways. The first is the MyD88-dependent pathway, which mirrors that of TLR2, leading to activation of NF-κB and MAPKs/AP-1, and the subsequent production of pro-inflammatory cytokines. The second is the MyD88-independent pathway, in which TLR4 recruits TRAM and then TRIF, initiating TRIF-dependent signaling. This results in activation and nuclear translocation of IRF3, which drives transcription of IFN-β (Gomes et al., 2012; Oliveira et al., 2008; Campos et al., 2004). Additionally, *Brucella* gDNA activates TLR9, triggering NF-κB and MAPK pathways via a MyD88-dependent mechanism, similar to TLR2 signaling (Oliveira et al., 2008; Gomes et al., 2012). TLR9 also recruits IRAK1/4 and TRAF6 through MyD88, which leads to activation of IRF7 and induction of IFN-α/β (Oliveira et al., 2008). Notably, although the non-classical LPS of *Brucella* elicits a weak immune response, other components such as OMPs and gDNA are more immunostimulatory. In particular, the recognition of *Brucella* gDNA by host TLR9 has been shown to trigger a strong immune response. gDNA from *Brucella* activates cGAS, while cyclic di-GMP (c-di-GMP) released by the bacterium activates STING, leading to STING- and IRF3-dependent IFN-β production (Costa Franco et al., 2018; Burdette et al., 2011). Autocrine signaling through the IFN I receptor (IFNAR) leads to activation of signal transducers JAK and STAT1, forming a heterotrimeric transcription factor (STAT1-STAT2-IRF9), which binds to IFN-stimulated response elements (ISREs) in the promoters/enhancers of IFN-stimulated genes (ISGs). This binding upregulates the expression of ISGs such as *Mx*, *OAS*, *PKR*, and *ISG15* (Gomes et al., 2012; Freitas et al., 2021; Szczerba et al., 2022; Samsing et al., 2020; Barrat et al., 2019). Activation of IFNAR can also induce the expression of GBPs, which facilitate the rupture of BCVs and promote the formation of inflammasomes, resulting in the release of IL-1β (detailed in the section on 5.3 Inflammasomes and pyroptosis pathways) (Marinho et al., 2024). Upon infection with *Brucella*, the nucleotide oligomerization domain-like receptor protein 3 (NLRP3) inflammasome is triggered by DAMPs, while the AIM2 inflammasome is activated by *Brucella* gDNA. Both inflammasomes facilitate the secretion of IL-1β and IL-18, triggering pyroptosis (Tupik et al., 2020; Figure 2).

## 5 Key cellular pathways of *Brucella*-mediated manipulation of host innate immunity

### 5.1 Selective subversion of autophagy pathways

Autophagy is a cellular stress response mechanism that involves engulfing and degrading intracellular components, including damaged organelles (e.g., mitophagy, ER-phagy, and ribophagy), protein aggregates (aggrephagy), and pathogens (heterophagy), to maintain the normal functions of cells. Initially, the substrate is encapsulated in a double-membrane structure to form autophagosomes, which are then transported to lysosomes to form degradable autophagolysosomes. Once the autophagic contents have been degraded, the products within autophagolysosomes are released, leading to the re-formation of lysosomes (Li J. et al., 2022). *Brucella* subverts the autophagy of its host cell to create a safe replication compartment, utilizing specific autophagic proteins. Several autophagic proteins are known to be involved in different stages of the intracellular cycle of *Brucella*. For example, Atg9 and WIPI1 participate in rBCV formation, while ULK1 and Beclin 1 mediate aBCV formation. Thus, *Brucella* has evolved to interfere with autophagy processes and thereby selectively subvert the autophagy of its host while establishing an intracellular niche for long-term replication and survival (Louche et al., 2023; Dong et al., 2023).

*Brucella* thrives in cells, triggering starvation-induced autophagy by competing for nutrients. Sentrin/SUMO-specific protease (SENP) negatively regulates starvation-induced autophagy. When starvation-induced autophagy occurs, SENP3 translocates from its nucleolar location to the cytoplasm, de-SUMOylating BECN1 to prevent its interaction with other autophagy proteins (including UVRAG, PIK3C3, and ATG14) and thereby downregulating autophagy (Liu et al., 2020). S2308 produces two effector proteins: NyxA, which enters host cells such as RAW264.7 cells and HeLa cells via the T4SS, and NyxB, whose entry route remains unclear. Both effectors retain SENP3 in the nucleus, with its resulting absence in the cytoplasm preventing the deSUMOylation of BECN1. Cytoplasmic PIAS3 can SUMOylate BECN1, promoting the formation of *Brucella*-induced foci (Bif), which contain ribosomal protein L5 (RPL5), the AAA-ATPase NVL, and NyxA/B near the bacteria. Bif components RPL5 and NVL are ribosomal biogenesis-associated proteins that promote rBCV formation and thus facilitate *Brucella* replication (Louche et al., 2023; Liu et al., 2020).

Previous research on the intracellular cycle of *Brucella* indicates that ULK1- and Beclin1-dependent autophagic nucleation contributes to the formation of aBCVs. These vacuoles allow the bacterium to exit its host cells, thereby completing its intracellular life cycle and enabling intercellular transmission (Dong et al., 2023). The formation of aBCVs involves a specific subset of autophagy-related molecular mechanisms rather than the entirety of classical autophagy. The effector protein BtpB, secreted by *Brucella* via the T4SS, inhibits autophagy and autophagolysosome formation through an unclear molecular mechanism (Li J. et al., 2022). Autophagy can be activated during the *Brucella*-induced UPR (Smith et al., 2013; Ogata et al., 2006). The VceA protein, secreted by *Brucella* via the T4SS, can suppress autophagy. Zhang et al. Zhang et al. (2019) demonstrated that, compared with the *VceA* knockout mutant (S2308Δ*VceA*), S2308 harboring the wild-type *VceA* gene, upon *in vitro* infection of HPT-8 cells, led to reduced expression levels of autophagy-promoting factors *Atg5* and *LC3-II*, while increasing the expression of autophagy-inhibitory markers *p62* and *LC3-I*. Furthermore, Wang et al. Wang et al. (2021a) showed that Omp31 from *B. melitensis* can induce autophagy in BV-2 microglial cells. This autophagic process suppresses phosphorylation of the NF-κB p65 protein at Ser536, reduces the nuclear translocation of phosphorylated p65 (p-p65), and decreases IκBα protein levels. As a result, activation of the NF-κB signaling pathway is blocked, leading to reduced production of TNF-α. Therefore, *Brucella* both exploits a subset of the autophagy machinery for aBCV formation and suppresses autophagy, aiding its release from host cells and preventing its killing and clearing by autophagy, which greatly benefits its intracellular survival and intercellular spread.

### 5.2 ER stress pathways

The ER is involved in the biosynthesis, folding, and modification of soluble and membrane proteins, while also playing a role in maintaining calcium homeostasis. When cellular physiological perturbations disrupt these ER functions, unfolded or misfolded proteins accumulate in the ER lumen. ER stress arises from overwhelm or impairment of protein folding capacity (de Jong et al., 2013). The UPR is triggered to restore cellular homeostasis, but may lead to apoptosis if unresolved (Luizet et al., 2021). In mammalian cells, the UPR is activated by three ER sensors: IRE1, protein kinase RNA (PKR)-like ER kinase (PERK), and activating transcription factor 6 (ATF6) (Taguchi et al., 2015). Smith et al. Smith et al. (2013) demonstrated that *B. melitensis* infection in mouse macrophages activated all three UPR pathways. However, in another study, *B. abortus* infection in HeLa cells led to time-dependent activation of the UPR exclusively through the IRE1 pathway (Taguchi et al., 2015). This discrepancy may be explained by differences between macrophages and epithelial cells.

*B. melitensis*-secreted TcpB induces the upregulation of host UPR-related genes and the remodeling of ER structure in mouse macrophages (e.g., RAW264.7 cells and BMDMs), leading to ER stress. The fusion of late eBCVs with the ER to form rBCVs in macrophages disrupts ER structure, leading to its re-formation, and thereby impairs ER homeostasis and induces ER stress (Smith et al., 2013). Under normal physiological conditions, IRE1, PERK, and ATF6 sensors are usually bound to GRP78/BIP in an inactive state. Both VceA and VceC reside in the ER, but only VceC induces ER stress by altering ER structure. VceC binds to GRP78/BIP, causing the release of GRP78/BIP from IRE1α and thereby activating the UPR (Zheng et al., 2024). Meanwhile, IRE1, aided by HeLa cell-derived Yip1A, forms a high-order complex at the ERES and undergoes autophosphorylation, leading to its activation. Activated IRE1 initiates the formation of ER-derived autophagic vesicles, with autophagy-related proteins Atg9 and WIPI1 recruited to the isolation membrane of ERES, independent of double FYVE domain containing protein 1 (DFCP1), in stark contrast to the mechanism of classical autophagy. The COPII vesicle components Sar1, Sec23, and Sec24 are upregulated to enhance the export of COPII vesicles through ERES. In addition, these vesicles serve as the structural core and membrane source for autophagosomes. Subsequently, they fuse with endosomal/lysosomal vesicles (produced upon the action of late eBCVs with lysosomes) to form rBCVs with ER membrane and self-replicating capabilities (Taguchi et al., 2015). Moreover, studies in mouse models and *in vitro* cell experiments have demonstrated that *Brucella* utilizes its T4SS-secreted effector proteins to activate IRE1α and its associated kinases ASK1 and JNK, thereby subverting the IRE1α-ULK1 signaling axis. This process involves the following steps: (1) *Brucella* enters host cells and is trafficked to the ER, inducing the host UPR directly or indirectly, and activating IRE1α signaling; (2) activated IRE1α initiates the activation of IRE1α-associated kinases, including ASK1 and JNK; (3) ASK1 and JNK drive the activation or assembly of downstream proteins ULK1, Atg9a, WIPI1, and Beclin1; and (4) these autophagic proteins drive membrane remodeling to promote the formation of intracellular rBCVs, thereby facilitating the intracellular parasitism of *Brucella* (Pandey et al., 2018). Therefore, UPR and autophagy are closely linked, jointly facilitating rBCV formation.

### 5.3 Inflammasomes and pyroptosis pathways

Inflammasomes are intracellular multi-protein complexes that play a key role in the innate immune response (Martinon et al., 2002). The main components of the classical inflammasome include receptor proteins (e.g., NLRP3 and AIM2), the adaptor protein apoptosis-associated speck-like protein-containing CARD (ASC), and the effector protein caspase-1 (Fu and Wu, 2023; Rathinam and Fitzgerald, 2016). When cells are stimulated by PAMPs, DAMPs, or other stimuli, receptor proteins are activated to interact with ASC through their specific structural domains, recruiting procaspase-1 to form inflammasomes (Fu and Wu, 2023). The activated inflammasomes mediate the self-cleavage and activation of procaspase-1; activated caspase-1 then cleaves the cytokines pro-IL-1β and pro-IL-18 (generated by TLR signaling) into mature, active IL-1β and IL-18, which are released from the cells to trigger inflammatory responses. Additionally, caspase-1 cleaves gasdermin D (GSDMD), releasing its NTD, which then oligomerizes and forms pores in the plasma membrane. These pores disrupt cellular osmotic balance, ultimately causing cell lysis and pyroptosis. Pyroptosis, in turn, leads to the release of cellular contents, including cytokines such as IL-1β and IL-18, which further exacerbate the inflammatory response (Rathinam et al., 2012; Tupik et al., 2020; Xu et al., 2020).

*B. abortus* infection triggers a series of intracellular changes, including P2 × 7 receptor-independent outflow of K^+^, production of mitochondrial ROS rather than NADPH oxidase-derived ROS, acidification of lysosomes, and release of cathepsin B into the cytoplasm, thereby activating the NLRP3 inflammasome (Campos et al., 2019). In addition, studies have shown that expression of GBP1-3, GBP5, and GBP7 in mice is significantly upregulated 4 h after infection with *B. abortus*, with GBP5 expression beginning to rise as early as 2 h post-infection and remaining the highest among all GBPs at each time point. These GBPs contribute to the rupture of BCVs, leading to the release of bacterial components such as gDNA and LPS. The released gDNA of *B. abortus* acts as a PAMP, which is recognized by and binds to AIM2, thereby activating the AIM2 inflammasome. This process is dependent on GBP5 and GBP2. Meanwhile, LPS is sensed via GBP5 to activate the non-canonical inflammasome pathway (Marinho et al., 2024; Figure 2). *Brucella* gDNA, which is recognized as a PAMP, binds to AIM2 to induce the production of AIM2 inflammasomes. Activation of the NLRP3 and AIM2 inflammasomes ultimately leads to the production of IL-1β and IL-18, which in turn recruit immune cells to trigger immune and inflammatory responses. In severe cases of infection, these pro-inflammatory cytokines induce the pyroptosis of inflammatory cells, thereby enhancing bacterial clearance (Tupik et al., 2020; Figure 2). Cerqueira et al. Cerqueira et al. (2018) revealed that the LPS of *B. abortus* triggers caspase-11/GSDMD-dependent pyroptosis of non-classical inflammasomes, thereby controlling infection and recruiting or activating immune cells. In contrast, Tupik et al. (2020) demonstrated that caspase-11 played a negligible role in *B. abortus*-infected cells. Nevertheless, *Brucella* has evolved to suppress inflammasome activation. As a member of the α-Proteobacteria, *Brucella* produce NO by converting nitrate to nitrogen via specific enzymes. NO represses the activity of caspase-1, thereby suppressing the production of IL-1β, IL-18, and GSDMD, curbing inflammation, and preventing pyroptosis. Additionally, NO blocks the assembly of the NLRP3 inflammasome through the nitrosylation of thiols (Campos et al., 2019). During *Brucella* infection, inflammasomes play a dual role in the dynamic interplay between the host immune response and the pathogen. On one hand, inflammasomes such as NLRP3 and AIM2 can be activated by *Brucella*, leading to the release of pro-inflammatory cytokines like IL-1β and IL-18, as well as the induction of pyroptosis. These mechanisms work together to enhance the host’s ability to clear the bacteria and positively regulate innate immune responses. On the other hand, *Brucella* can counteract inflammasome activation by producing NO, which inhibits Caspase-1 and related pathways. The intricate interaction reflects a complex immune balance, shaped by both host defenses and bacterial adaptation strategies, highlighting the inflammasome’s central role in immune regulation throughout the course of infection.

### 5.4 Apoptosis pathways

Both apoptosis and pyroptosis are triggered by caspases, although the specific caspases involved vary. Caspases can initiate programed cell death in infected cells, and the resulting cell debris is subsequently cleared by phagocytes, contributing to the elimination of invading bacteria. However, *Brucella* evades the host immune response by precisely modulating the apoptotic process. This regulation creates a favorable intracellular environment for the bacteria’s survival and replication. The mechanisms employed by *Brucella* to control host cell apoptosis are notably complex and diverse. *Brucella* infection triggers the expression of A20, a zinc finger protein, in macrophages (Guo et al., 2023). A20, or TNF-α-induced protein 3 (TNFAIP3), is an enzyme that is proposed to deubiquitinate key intermediates in the NF-κB signaling pathway, including receptor-interacting protein (RIP)1, RIP2, TNF receptor-associated factor 6 (TRAF6), and mucosa-associated lymphoid tissue lymphoma translocation gene 1 (MALT1). This deubiquitination disrupts specific protein–protein interactions, thereby repressing NF-κB activity and consequently modulating the functionality, proliferation, and differentiation of various immune cells, including T cells, B cells, macrophages, and DCs (Zhang M. et al., 2016; Harhaj and Dixit, 2012). Furthermore, A20 may protect intestinal epithelial cells, embryonic fibroblasts, hepatocytes, pancreatic β cells, T cells, NK cells, and macrophages against TNF-induced apoptosis, thereby indirectly repressing inflammation (Guo et al., 2023; Priem et al., 2020).

ER stress is a critical pathway leading to apoptosis. As previously mentioned, *Brucella* infection induces ER stress. Persistent or excessive ER stress that surpasses the capacity of the UPR to manage misfolded or unfolded proteins leads to upregulation of C/EBP-homologous protein (CHOP) expression, which subsequently activates downstream gene expression, culminating in apoptosis. VceC functions bidirectionally. On one hand, VceC binds to GRP78/BIP, activating the IRE1 pathway and regulating the ER stress response. On the other hand, VceC upregulates GRP78/BIP expression to aid in coping with ER stress and suppresses CHOP expression to reduce ER stress-induced apoptosis, thereby supporting intracellular replication of *B. suis* in goat trophoblast cells. In addition, VceC has been shown to potentially collaborate with LPS to block CHOP-mediated apoptosis, aiding the persistence of *Brucella* in goat trophoblasts. However, this mechanism requires further validation (Cao and Kaufman, 2012; Zheng et al., 2024; Zhi et al., 2019). Conversely, Byndloss et al. (2019) demonstrated that VceC induced CHOP expression in the trophoblasts of *B. abortus*-infected mice, causing cell death, placental inflammation, and ultimately miscarriage.

Regarding the regulation of macrophage apoptosis, calpain 2 fosters macrophage apoptosis across diverse pathologies. Arsenic in renal macrophages raises intracellular calcium levels, leading to the activation of calpain 2, which in turn activates caspase 3 and promotes apoptosis (Banerjee et al., 2011). In the context of *Brucella*, studies have shown that infection with S2308 (a smooth strain) induces an increase in intracellular calcium in macrophages, which in turn activates the calcium-dependent E3 ubiquitin ligase Nedd4. Nedd4 can suppress the apoptosis of macrophages by ubiquitinating calpain2 and blocking the activation of apoptotic effector caspase3 (Cui et al., 2014). In contrast, RB51 (a rough strain) triggers caspase 2-mediated apoptosis in macrophages, through a process linked to the activation of NF-κB and the production of inflammatory cytokines (e.g., TNF-α). The differences in how the two *Brucella* strains regulate macrophage apoptosis are directly related to the function of the O antigen. Studies have confirmed that the O antigen of smooth *Brucella* strains exerts an anti-apoptotic effect within monocytes and macrophages. In contrast, rough strains lack a complete O antigen and therefore exhibit a pro-apoptotic phenotype. This may help explain why smooth strains are generally more virulent than rough strains (Fernandez-Prada et al., 2003). Interestingly, both S2308 and RB51 can induce caspase 2-dependent apoptosis in DCs, with S2308 having a stronger effect. In summary, S2308 infection of DCs enhances apoptosis, impedes maturation, and lowers their capacity to present the S2308 antigen to T cells. Additionally, S2308 suppresses macrophage apoptosis, allowing the bacteria to avoid exposure to a hostile extracellular environment and facilitating the replication and survival of *Brucella* in macrophages. The induction of DC death and the suppression of macrophage apoptosis represent mechanisms that may contribute to the pathogenicity of *Brucella* (Gomes et al., 2012). It has been shown that the *Brucella* T4SS effector protein BtpB causes DNA fragmentation and induces macrophage apoptosis, although its precise mechanism remains unknown (Li J. et al., 2022). With respect to the functional diversity of effector proteins, Zhang et al. (2019) found that, compared with VceA mutants, the wild-type VceA protein increased the mRNA expression of caspase-3 (*Casp3*) while decreasing that of B-cell lymphoma 2 (*Bcl-2*). Given that *Casp3* is an apoptosis-promoting gene and *Bcl-2* is an apoptosis-resistance gene, it can be inferred that VceA promotes apoptosis. Also, Omp31 has been shown to inhibit host cell apoptosis. Zhang K. et al. (2016) demonstrated that infection with wild-type *B. melitensis* 16M, but not with an Omp31-deficient mutant, markedly reduced apoptosis in RAW264.7 macrophages, suggesting that Omp31 plays a critical role in modulating host cell death responses. The mechanism is as follows: binding of TNF-α to TNFR-1 activates caspase-8, which in turn cleaves and activates the executioner caspase-3, leading to apoptosis. TNF-α also indirectly regulates Bcl-2 family members by upregulating the pro-apoptotic protein Bax and downregulating the anti-apoptotic protein Bcl-2. This alters mitochondrial membrane permeability, facilitating the release of cytochrome c (Cyt c) into the cytoplasm. Cyt c then activates caspase-9, which also activates caspase-3 and induces apoptosis. Omp31 inhibits the expression of TNF-α and thereby blocks both apoptotic pathways, effectively preventing cell death. Studies have shown that *M. tuberculosis* suppresses host cell apoptosis during the early stages of infection, thereby creating a favorable intracellular environment for its survival and replication. In the later stages, TNF-α-induced apoptosis may facilitate the release of the pathogen from host cells, promoting its spread to neighboring cells. As a facultative intracellular bacterium like *M. tuberculosis*, *Brucella* may adopt similar strategies to modulate host cell fate and sustain its infectious cycle (Rodríguez-González and Gutiérrez-Kobeh, 2023). These findings collectively demonstrate that *Brucella* evades host immune responses and sustains intracellular survival by secreting various effector proteins and virulence factors, such as VceC and Omp31, and by regulating key host molecules including A20 and Calpain2. By selectively promoting or inhibiting apoptosis depending on the stage of infection and the type of host cell involved (such as macrophages, DCs, or trophoblasts), *Brucella* establishes persistent infection within the host.

### 5.5 Ferroptosis pathways

Characterized by iron-dependent membrane lipid peroxidation, ferroptosis is a novel form of programed cell death that is distinct from apoptosis, necrosis, and autophagy (Dixon and Olzmann, 2024). The mechanism of ferroptosis includes iron overload, increased ROS, and membrane lipid peroxidation. Specifically, Fe^3+^ in the blood circulation binds to transferrin for cellular entry via transferrin receptor 1 (TFR1)-mediated endocytosis (Parrow et al., 2019). Fe^3+^ is reduced to Fe^2+^ by six-transmembrane epithelial antigen of prostate 3 (STEAP3), and subsequently released from divalent metal-ion transporter 1 (DMT1)-containing endosomes into the cytoplasmic labile iron pool (Koskenkorva-Frank et al., 2013). Mitochondria generate ROS (e.g., superoxide anion [O_2_⋅^–^]). The disproportionation of O2⋅^–^ yields hydrogen peroxide, generating hydroxyl radical (⋅OH) molecules through a Fenton reaction with Fe^2+^ that attack phospholipids with polyunsaturated fatty acid chains (PUFA-PL) in cell membranes, causing peroxidation and forming PUFA-PL hydroperoxide (PUFA-PL-OOH) (Dixon and Olzmann, 2024). Under normal conditions, the lethal PUFA-PL-OOH is reduced by cellular antioxidant defense system pathways, which involve glutathione peroxidase 4 (GPX4)/glutathione (GSH), ferroptosis suppressor protein 1 (FSP1)/coenzyme Q10 (CoQ10)/NADPH, dihydroorotate dehydrogenase (DHODH), and GTP cyclohydrolase 1 (GCH1)/tetrahydrobiopterin (BH4). These pathways avoid the accumulation of PUFA-PL-OOH, thereby inhibiting the onset of ferroptosis (Jiang et al., 2021; Dixon and Olzmann, 2024). However, disordered iron metabolism (intracellular Fe^2+^ overload) and/or antioxidant defense dysfunction initiates lipid peroxidation, raising membrane tension and activating the piezo-type mechanosensitive ion channel component 1 (Piezo1) and transient receptor potential (TRP) pathways, which leads to ionic dysregulation, cell osmotic swelling, and plasma membrane rupture, ultimately inducing ferroptosis (Dixon and Olzmann, 2024). Lipid peroxidation results in the accumulation of toxic aldehydes including malondialdehyde (MDA) and 4-hydroxynonenal (4-HNE), which can also cause membrane rupture (Zhang et al., 2024).

Hu et al. (2023) discovered that infection of RAW264.7 macrophages with RB14, a rough mutant of *B. abortus*, induced ferroptosis, whereas infection with S2308 suppressed it. RB14, lacking the O antigen of LPS, is derived from the smooth parental strain. Considering that the commonly seen *Brucella* isolates (*B. abortus*, *B. suis*, and *B. melitensis*) are all smooth strains, Zhang et al. (2024) explored the manipulation of host cell ferroptosis by the smooth *B. melitensis* M5 during infection of RAW264.7 macrophages. This revealed that M5 inhibited ferroptosis via the GCH1/BH4 pathway in the early stage of infection, promoting its intracellular proliferation, but induced ferroptosis through the GPX4/GSH pathway to facilitate its escape and spread in the late stage of infection. Interestingly, M5 had no impact on the expression of FSP1 or DHODH. Furthermore, the intracellular replication of *B. melitensis* M5 was blocked by ferroptosis-inducing drugs and enhanced by ferroptosis inhibitors, implying that host cell ferroptosis restricts the intracellular survival of this pathogen (Zhang et al., 2024).

### 5.6 cGAS-STING pathways

Upon entry of *Brucella* into host macrophages, its gDNA activates the STING-TANK binding kinase 1 (TBK1) signaling cascade. As a key hub protein in the innate immune signaling pathway, TBK1 undertakes the signaling of TLRs, NOD-like receptors, and RIG-I-like receptors. As a key bridge connecting multiple PRRs, it plays a role in inducing the expression of type I IFN, while linking cGAS-STING signal transduction and activating the NF-κB pathway. In addition to cGAS, several other intracellular DNA sensors have been identified, including TLR9, AIM2, RNA polymerase III, DNA-dependent activator of IFN-regulatory factors (DAI), leucine-rich repeat in flightless-1 interaction protein 1 (LRRFIP1), IFN-inducible protein 204 (IFI204), meiotic recombination 11 homolog A (Mre11), DEAD-box RNA helicase-1 gene (Ddx41), and LSm14A. All of these sensors are capable of triggering innate immune responses (Costa Franco et al., 2018; Oliveira et al., 2008).

STING can be activated in two ways: (1) bacterial secretion of cyclic dinucleotides (CDNs), including cyclic-di-AMP by *Listeria monocytogenes* and cyclic-di-GMP by *Brucella*, which serve as direct activators of the STING pathway (Burdette et al., 2011; Costa Franco et al., 2018); and (2) binding of *Brucella* DNA to cGAS, leading to the synthesis of cyclic GMP-AMP (cGAMP) from the substrates GTP and ATP (Li X. et al., 2022; Costa Franco et al., 2018; Tong et al., 2024). This cGAMP binds to STING on the ER as a second messenger, resulting in a conformational change in STING that activates it (Costa Franco et al., 2018; Ishikawa et al., 2009; Chen et al., 2016; Tong et al., 2024). Activated STING then transits from the ER to the ERGIC and Golgi, during which the carboxy-terminus of STING recruits TBK1, activating it to phosphorylate the transcription factor IRF3. Dimerized, phosphorylated IRF3 enters the nucleus as a transcription factor, inducing IFN-β expression (Costa Franco et al., 2018; Ishikawa et al., 2009; Chen et al., 2016). The signaling triggered by the binding of IFN-β to IFNAR positively regulates the expression of GBPs. This regulation is not an isolated event, but instead involves coordinated interactions with the IRF1 and STING pathways, together forming a complex regulatory network that precisely controls GBP expression (Costa Franco et al., 2018; Figure 2). Subsequently, GBPs target the BCV, disrupting its membrane integrity and inducing lysis. This process leads to the release of PAMPs (e.g., gDNA and c-di-GMP) into the host cell cytoplasm (Meunier et al., 2015; Costa Franco et al., 2018). The PAMPs *Brucella* gDNA and cyclic-di-GMP activate the cGAS-STING and STING pathways, respectively, inducing the production of type I IFN (Costa Franco et al., 2018). Additionally, STING initiates the activation of the inhibitor of NF-κB kinase (IKK), resulting in the phosphorylation and ubiquitination of the inhibitory protein IκB associated with NF-κB, ultimately facilitating the proteasomal degradation of IκB. Subsequent to its dissociation from the NF-κB/IκB complex, NF-κB translocates to the nucleus, where it collaborates with transcription factors (e.g., IRF3) to modulate gene transcription, inducing the expression of IFN and pro-inflammatory cytokines, such as TNF-α, IL-1β, and IL-6 (Chen et al., 2016; Figure 2). The cGAS-STING pathway also elicits the production of cytokines (e.g., IFN-I, TNF-α, IL-1β, and IL-6) to counteract *Brucella*. During *Brucella* infection, STING also exerts antibacterial effects by regulating hypoxia-inducible factor 1-alpha (HIF-1α)-mediated metabolic reprograming in macrophages. Specifically, STING activation increases intracellular succinate levels and mitochondrial reactive oxygen species (mROS) production, which promotes the stabilization of HIF-1α. Stabilized HIF-1α facilitates M1 polarization of infected macrophages, enhances the expression of glucose transporter 1 (GLUT1) to increase glucose uptake, induces NO production, activates the inflammasome, and promotes the release of IL-1β. In addition, HIF-1α reshapes the metabolic profile of macrophages by enhancing glycolysis and suppressing oxidative phosphorylation (OXPHOS)—a metabolic shift that is key to IL-1β production. This HIF-1α–driven proinflammatory phenotype and metabolic reprograming together contribute to the control of *Brucella* replication, helping the host eliminate intracellular bacteria (Gomes et al., 2021).

However, *Brucella* has developed mechanisms to manipulate such an innate immune response. *Brucella* Omp25 specifically targets the cGAS-STING signaling pathway, inhibiting its production of IFN-β, TNF-α, and IL-12 by promoting cGAS degradation in various mammalian monocytes/macrophages via the ubiquitin-proteasome-dependent pathway (Li et al., 2021). In addition, *Brucella* can utilize its T4SS to upregulate miR-24, a microRNA that targets STING mRNA. The elevated level of miR-24 leads to reduced STING protein expression, thereby disrupting the host’s cytosolic immune surveillance and facilitating *Brucella* survival (Khan et al., 2020). In summary, the host mounts an immune defense against *Brucella* infection primarily through the cGAS-STING signaling pathway, which promotes the release of pro-inflammatory cytokines and triggers HIF-1α–mediated metabolic reprograming in macrophages. However, *Brucella* counteracts this defense by degrading cGAS and downregulating STING protein expression. The outcome of this immune tug-of-war determines whether the intracellular infection is successfully cleared or progresses into chronic infection.

## 6 Immune dynamics in a murine model of *Brucella* infection

Murine brucellosis is typically divided into four stages: the initial infection stage (≤ 48 h), the acute phase (from day 3 to weeks 2–3 post-infection), the chronic stable phase (weeks 3 to 8–11), and the chronic declining phase (beyond 36 weeks) (Grilló et al., 2012).

During the early stage of infection, the innate immune response is not strongly activated. This is evidenced by normal counts of blood cells and platelets, a lack of pro-inflammatory cell recruitment at the infection site, and minimal serum levels of cytokines such as IL-1β, TNF-α, IL-10, and IL-6. Chemokines like MCP-1 and RANTES also remain at very low concentrations. In addition, there is no evidence of fibrinogen synthesis or degradation, nor are there signs of coagulation abnormalities. At this stage, *Brucella* can already be detected in macrophages. *Brucella* is detectable in the liver within just 3 h post-inoculation (Barquero-Calvo et al., 2007; Grilló et al., 2012). Despite their presence, *Brucella* barely activates the complement system, and macrophages exhibit limited bactericidal activity. Tissue damage caused by the bacteria is also minimal at this point (Barquero-Calvo et al., 2007).

In the acute phase, *Brucella* rapidly replicate within target cells such as macrophages, DCs, or trophoblasts. Meanwhile, both innate and adaptive immune responses are activated. Innate immune cells, including macrophages, neutrophil, and other cells, participate in the host’s defense. A type IV delayed-type hypersensitivity (DTH) reaction is also triggered, marking the initiation of a Th1-type immune response. By 1 week post-infection, high levels of cytokines such as IFN-γ, IL-12, IL-6, and RANTES can be detected in the serum of susceptible mice. However, some cytokines remain at relatively low levels. For example, in the acute phase, the production of IL-18, which works together with IL-12 to induce the production of IFN-γ, is suppressed in the splenocytes of mice infected with *B. abortus*. Additionally, the acute phase is marked by significant inflammatory responses, including splenitis, lymphadenitis, and granuloma formation in the liver (Grilló et al., 2012).

During the chronic stable phase, the bacterial load in the target organs reaches its peak and remains at a high level, establishing a stable plateau. In this phase, granulomas in the liver, bone marrow, and spleen are most prominent. The production of IFN-γ in susceptible mice significantly decreases in this phase. Serum levels of IL-6 peak at the end of the acute phase and remain relatively high during the first 2 weeks of the chronic stable phase. In contrast, the secretion of IL-2, IL-10, GM-CSF, and IL-4 is minimal during this period (Grilló et al., 2012).

During the chronic regression phase, the bacterial load in the target organs gradually decreases, and the splenomegaly phenomenon also progressively alleviates. Pathological damage in the liver and spleen gradually disappears. DTH weakens, and the majority of cytokines in the blood decrease, with some even becoming undetectable. However, immune memory seems to be fully consolidated (Grilló et al., 2012).

Notably, murine models are not the natural hosts of *Brucella*, and the pathogenesis of brucellosis in these models may differ from that in humans in certain respects, such as the pathological features of chronic infection and the dynamics of immune responses. Despite this limitation, murine models remain the most widely used experimental system for studying *Brucella* immune evasion mechanisms and evaluating vaccine efficacy.

## 7 Innate and adaptive immunity in host defense against *Brucella*

The innate defenses against *Brucella* include physical barriers on the body surface, PMNs, macrophages, DCs, NK cells, chemokines, PRRs, and the complement system. Later, *Brucella* infection elicits three primary adaptive immune responses: (1) CD4^+^, CD8^+^, and γδ T cells secrete IFN to activate the bactericidal functions of macrophages, preventing the intracellular survival of the bacteria; (2) cytotoxic CD8^+^ T cells target the infected macrophages; and (3) Th1 antibody subtypes (e.g., IgG2a and IgG3) enhance macrophage phagocytosis via ADCP, aiding in *Brucella* elimination while also facilitating its invasion of phagocytic cells, which it uses as a base for replication and survival (Guo et al., 2023).

**FIGURE 4:**
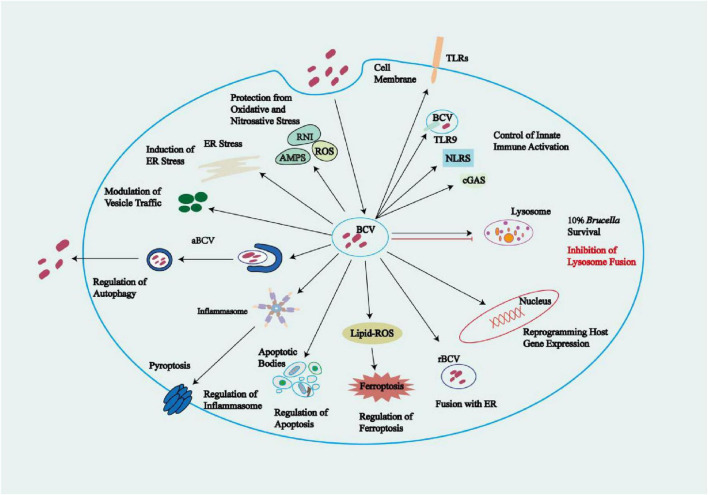
Manipulation of host innate immunity by *Brucella*. Upon recognition of *Brucella* by cell membrane or endosomal receptors, host cells employ a variety of mechanisms to kill the pathogen, including bacterial compartmentalization, oxidative and nitrosative stress, antimicrobial peptides, lysosome-mediated degradation, autophagy, inflammasome activation, pyroptosis, apoptosis, and ferroptosis. *Brucella* can regulate host receptor-activated signaling pathways, interact with endocytosis pathways to evade phagolysosome fusion, and manipulate the mechanisms underlying vesicle trafficking, inflammasome activation, apoptosis, ferroptosis, and autophagy, thus creating favorable conditions for its survival. AMPs, antimicrobial peptides; NLRs, NOD-like receptors; RNLs, retinoic acid-inducible gene I (RIG-I)-like receptors.

In conclusion, *Brucella* infection induces weak innate immune responses in humans. *Brucella* employs a variety of strategies to suppress innate immunity: blockage of TLR/NLR signaling pathways and the complement system; disruption of pyroptosis, apoptosis, and ferroptosis; induction of ER stress; modulation of autophagy; neutralization of antimicrobial peptides, oxidative stress, and nitrosative stress; regulation of vesicle trafficking; reprograming of host nuclear gene expression; and prevention or suppression of BCV-lysosome fusion (Figure 4). The successful implementation of these measures by *Brucella* significantly suppresses host innate immunity, initiating a cascade of reactions. On the one hand, the decreased secretion of host pro-inflammatory cytokines and chemokines leads to reduced recruitment and activation of macrophages, DCs, and other phagocytes at the infection site; on the other hand, the impaired phagocyte function contributes to inadequate activation of CD8^+^ T lymphocytes (which are responsible for cellular immunity), culminating in adaptive immunosuppression. In such an immunosuppressed environment, *Brucella* can thrive and smoothly transition to the chronic phase. The intracellular niche favors *Brucella* replication and survival, while inadequate immune protection leads to prolonged tissue and organ invasion, causing chronic inflammation and jeopardizing the normal functions of the body.

## 8 Summary and outlook

*Brucella* has co-evolved with its hosts over millions of years, developing a range of virulence strategies that facilitate its survival and ultimately lead to disease onset through the manipulation of host systems. *Brucella* employs several primary strategies to evade the host immune system, enabling its proliferation and sustained intracellular survival, which leads to chronic infection. These include the following: inhibition of complement and TLR pathways; blockage of maturation and disruption of antigen presentation by DCs; formation of intracellular replication vesicles; and manipulation of autophagy, ER stress, inflammasome activation, pyroptosis, apoptosis, ferroptosis, and the cGAS-STING pathway, among others. *Brucella* produces virulence factors and effector proteins that manipulate host cell signaling pathways, disrupting normal cellular processes to its advantage. However, the mechanisms by which some effector proteins regulate cellular processes during infection are poorly understood. Autophagy and programed cell death pathways (e.g., pyroptosis, apoptosis, and ferroptosis) serve as host defense strategies, containing infections by eliminating intracellular pathogens from infected cells (Zhang et al., 2024). However, *Brucella* can exploit programed cell death pathways to evade killing, employing specific molecular mechanisms to enhance its replication, survival, and dissemination within host cells. Therefore, researchers can disrupt the specific molecular mechanisms by which *Brucella* manipulates autophagy, pyroptosis, apoptosis, and ferroptosis, enabling the corresponding cell death program to proceed as normal and thereby eradicating the bacteria. Doxycycline (Dox) can inhibit CALR protein expression, activate the JNK/p53 signaling pathway, and induce apoptosis in HMC3 cells, thereby achieving the therapeutic goal of treating brucellosis (Wang et al., 2021b). The interaction between *Brucella* and programed cell death remains unclear. Thus, elucidation of the mechanisms governing these interactions is essential for identifying potential drug targets that could induce programed cell death and eradicate the pathogen.

It is noteworthy that *Brucella* adopts significantly different survival strategies in various host cells: in non-phagocytic cells, early eBCV can avoid degradation by late endosomes and lysosomes through specific mechanisms; however, in phagocytic cells (such as macrophages), early eBCV briefly fuses with late endosomes and lysosomes. Subsequent studies, however, have revealed two key mechanisms that effectively block the fusion of eBCV with lysosomes: *Brucella* activates the host RIDD pathway to disrupt BLOS1-mediated immune defense and secretes CβGs. This leads to a paradoxical conclusion in research: in phagocytic cells, it remains unclear whether both the fusion of early eBCV with lysosomes and the anti-fusion mechanisms coexist simultaneously, or if only one of these processes actually occurs. Further studies are needed to clarify the true nature of these seemingly contradictory phenomena in macrophages. In addition, compared with other intracellular bacteria of the *Proteobacteria*, the number of identified *Brucella* effector proteins remains relatively limited. This suggests that many effectors may still be undiscovered. Therefore, further in-depth research is needed in this area, as effector proteins are key molecules through which *Brucella* evades host immune responses.

In summary, future research need to focus on the molecular mechanisms by which *Brucella* regulates programed cell death, the differences in vesicle trafficking across various cell types, and the identification of novel effector proteins. These efforts will be essential for uncovering the mechanisms underlying its intracellular survival and for developing targeted strategies for prevention and treatment.
