# Correction: Beyond survival to domination: *Brucella's* multilayered strategies for evading host immune responses

**DOI:** 10.3389/fmicb.2025.1669739

**Published:** 2025-09-02

**Authors:** Zhimeng Wei, Shuai Zhang, Xingya Wang, Jie Bai, Hui Wang, Yuanchao Yang, Jingbo Zhai

**Affiliations:** ^1^School of Basic Medical Sciences, Inner Mongolia Minzu University, Tongliao, China; ^2^Department of Clinical Laboratory, Keerqin District First People's Hospital, Tongliao, China; ^3^Department of Polyclinics, Tongliao City Center for Disease Control and Prevention, Tongliao, China; ^4^Brucellosis Prevention and Treatment Engineering Research Center of Inner Mongolia Autonomous Region, Tongliao, China; ^5^Key Laboratory of Zoonose Prevention and Control at Universities of Inner Mongolia Autonomous Region, Tongliao, China

**Keywords:** *Brucella*, virulence factors, immune escape, innate immunity, inflammasomes, ferroptosis

During the production process, a reference that was missing from the reference list was incorrectly added to an inappropriate position in the main text, causing multiple references to shift by one position overall and resulting in misalignment of the in-text citations.

The reference for [Bhardwaj et al., 2021] was erroneously written as Bhardwaj, A., Sita, K., Sehgal, A., Bhandari, K., Kumar, S., Prasad, P., et al. (2021). Heat priming of lentil (Lens culinaris Medik.) Seeds and foliar treatment with γ- aminobutyric acid (GABA), confers protection to reproductive function and yield traits under high-temperature stress environments. *Int. J. Mol. Sci*. 22:5825. doi: 10.3390/ijms22115825.

It should be Xiong, X., Li, B., Zhou, Z., Gu, G., Li, M., Liu, J., et al. (2021). The VirB system plays a crucial role in *Brucella* intracellular infection. *Int. J. Mol. Sci*. 22:13637. doi: 10.3390/ijms222413637.

The original version of this article has been updated.

